# Granzyme B, HIF-1α, VEGFA and SerpinB9 in the local tumour immune system of immune-stimulated slice cultures of head and neck cancer

**DOI:** 10.1186/s12885-026-16282-x

**Published:** 2026-06-11

**Authors:** Maria do Carmo Greier, Jozsef Dudas, Julia Federspiel, Federico Ferro, Phillipp Bergher, Benedikt Gabriel Hofauer

**Affiliations:** Department of Otorhinolaryngology, Head and Neck Surgery, Medical University of Innsbruck, Innsbruck, Austria

**Keywords:** Heterogeneity, Immune escape, Antibody beads, Immune infiltrate, Hypoxia

## Abstract

**Background:**

Even when T-cell activation is successful, head and neck squamous cell carcinoma (HNSCC) often becomes resistant to immunotherapy. This study investigated the molecular factors that influence immune-induced tumour cell death in organotypic HNSCC slice cultures (SCs).

**Methods:**

SC samples were obtained from 23 patients (22 with HNSCC and one tumour-free control). Immune stimulation was performed using a T-cell activator kit. The protein levels of granzyme B (GrB), cleaved caspase-3 (CC3), Slug, hypoxia-inducible factor-1 alpha (HIF-1α), vascular endothelial growth factor alpha (VEGFA) and SerpinB9 were assessed using immunohistochemistry (IHC) and immunofluorescence (IF). The patient samples were stratified according to Slug expression and HPV status. Slug, VEGFA, HIF-1α, HPV positivity and baseline SerpinB9 were considered input determinants, whereas GrB, CC3 and induced SerpinB9 were considered response factors.

**Results:**

The baseline intensity of HIF-1α was low in most tumour tissue samples, except in two patients who had high levels. SerpinB9 levels were higher in tumour cells from five patients than in a single tumour-free control sample. VEGFA immunofluorescence (IF) levels were clearly higher in several tumour samples, including HPV-positive cases, compared to the single tumour-free control sample. A significant correlation was observed between HIF-1α and VEGFA IF sum intensities. Following immune stimulation, the intensity of GrB increased in 14 out of 22 tumour samples. CC3 was induced in tumour cells in 12 out of 19 samples, particularly in five out of six HPV-positive samples, while SerpinB9 was induced in nine out of 18 samples. Interestingly, this occurred in only two out of six HPV-positive samples. The induction of CC3 and SerpinB9 was significantly correlated (*r* = 0.67, *p* < 0.01). No difference in GrB induction was observed between Slug-positive and Slug-negative tumours. However, lower levels of CC3 and SerpinB9 induction were observed in Slug-positive tumours.

**Conclusions:**

The Slug–HIF-1α–VEGFA axis may enable tumour cells to evade the primary immune response by inhibiting apoptosis induced by the immune system. Extensive immune activation induced both the caspase cascade and the protective factor SerpinB9 in tumour cells of HPV-negative HNSCC mainly. GrB, CC3 and SerpinB9 exhibited reduced spatial gradients at increasing distances from the immune-stimulating beads.

**Supplementary Information:**

The online version contains supplementary material available at 10.1186/s12885-026-16282-x.

## Background

### Head and neck squamous cell carcinoma

Head neck squamous cell carcinoma (HNSCC) tumours display high interpatient and intrapatient heterogeneity [1]. HNSCC patients have relatively low response rate for standard therapy. The low therapy response rate and tumour heterogeneity are related with each other, as heterogeneity generates multiple clones that survive the therapy. Tumour heterogeneity is regulated by the complex tumour microenvironment (TME), which is patient specific. The TME is composed of extracellular matrix, blood vessels immune cells and cancer associated fibroblasts (CAFs) [2, 3]. TME is involved in cancer progression by influencing the immune system, proliferation, and metastasis [4]. Therefore, the TME plays an important role in cancer immunosurveillance and immune escape.

### Anti-tumour immune response, anti-CD3/CD2/CD28 beads

The anti-tumour immune response relies on tumour-cell-killing activities of cytotoxic (CD8^+^) T-lymphocytes (CTLs) and on natural killer cells (NK-cells). T cells are activated when the T cell receptor (TCR) recognizes a peptide on an MHC (major histocompability complex) molecule. CD2 and CD3 associated with the TCR–peptide–MHC complex, are required to transmit activation signals [5, 6]. Moreover, costimulatory receptors like CD28 trigger T cell activation [7]. Therefore, anti-CD3/CD2/CD28 bead co-stimulation is widely used to activate and expand T cells [8, 9]. These beads enhance T cell proliferation, cytokine production, and cytotoxicity, triggering immune responses ex vivo [10]. This established test method serves as a valuable tool to study antitumour immunity [11]. 

Activated CD8^+^ T cells discharge cytolytic Granzyme B (GrB), which is stored in lytic granules. Release of GrB granules can lead to apoptosis of the target cell by activating a caspase cascade. Cleavage of caspase 3 produces its active form, the cleaved caspase 3 [12–14]. GrB is an indicator of cytotoxic T lymphocyte (CTL) activation, and a considerable marker for effective T cell function. The cytotoxic effect of GrB can be counteracted by SerpinB9, a serine protease inhibitor that binds GrB in the cytosol and prevents it from initiating apoptosis. SerpinB9 is positioned between the activated GrB and the caspase cascade. SerpinB9 acts as a pseudosubstrate for GrB and forms an irreversible complex with it [15]. By blocking GrB activity, SerpinB9 enables tumour cells to evade CTL- and NK cell–mediated killing, and has been identified as a key resistance factor in multiple tumour types [16].

As aforementioned, co-stimulation of CD3 and CD28 T-cell-receptors represents a significant approach of novel tumour immunotherapy [17]. Previous published evidence revealed that anti-CD2/CD3/CD28 co-stimulation leads to expansion of NK cells, which produce GrB, and have cytotoxic activity [18]. This means that multiplex immune stimulation, with expansion and activation of T lymphocytes and NK-cells is considered as a relevant path of current immunotherapy. 

### Immunosuppression and immune escape

HNSCC cells can evade surveillance by the host immune system using several mechanisms. One mechanism is TME remodelling. In the common immune exclusion phenotype within HNSCC, immune cells are restricted to the stroma and cannot infiltrate tumour cell nests due to barriers like extracellular matrix (ECM), lack of lymphatic vessels, and disorganized blood vessels. Conditions such as hypoxia, acidity, and nutrient deficiency further limit immune cell penetration and therefore have a direct effect on immune cell function [19, 20]. Hypoxia-inducible transcription factor (HIF) activated by decreased oxygen levels strongly influence immune cell function [21, 22]. HIF-1α can activate the expression of Vascular Endothelial Growth Factor alpha (VEGFA), which promotes angiogenesis. VEGFA also contributes to immune evasion by inhibiting the maturation and function of immune cells [23].

In the immune suppression and escape relationship the HIF-1α/VEGFA axis is an established and frequently discussed pathway, which was also in the focus of our investigations [24]. Interestingly, targeting HIF-1α with drugs is being explored as a potential treatment strategy [25]. Silibinin, a HIF-1α synthesis blocker has already achieved an approved clinical usage stage [25, 26].

### Microenvironment – model HNSCC - SC

While cell cultures serve as valuable models for studying metabolism and cellular functions of tumour and immune cells, they are limited in representing the three-dimensional interactions found in TME [27]. Over the past few years, new in vitro and ex vivo models have been created to better mimic the complex dynamics of the TME [28]. One such model involves cultivating tissue sections from patient tumour biopsies, known as slice cultures (SCs), which offers a more accurate representation of the TME compared to standard cell cultures [29]. The use of HNSCC SCs represents a clinical setting where fresh tumour samples are accessible. These cultures maintain viability, preserve the local microenvironment, and respond to immunological modifiers [30]. However, their availability is scarce, and advanced techniques like genetic modification are difficult to implement. While flow cytometry is a reliable method for characterizing cells, its application to slice cultures is problematic due to potential epitope loss during cell separation, which can compromise the results. As a result, histological analysis is typically the preferred approach for examining slice cultures. In our previous study, we successfully implemented a bead-based immunostimulation model in SC, which was originally established for use with peripheral blood mononuclear cells (PBMCs) [10, 31]. Here we investigated the role of patient-specific Slug/HIF-1α/VEGFA background for immune stimulation efficiency and immune-induced tumour cell death.

Our hypothesis was that, Slug related with HIF1-α, determine high VEGFA and SerpinB9 levels in HNSCC and contribute to immune suppressive TME. If the Slug- HIF1-α-VEGFA axis is upregulated in HNSCC tissue this inhibits either the activation of GrB in TME or the induction of apoptosis in tumour cells.

## Methods

### Study population and preparation of HNSCC slice cultures

This study included all consenting patients with newly diagnosed, locally advanced HNSCC treated at the Medical University of Innsbruck’s Department of Otorhinolaryngology – Head and Neck Surgery from January to September 2022. Patients must have been over 18, have undergone diagnostic endoscopy with anaesthesia and biopsy, and had a primary tumour classified as T3-T4. Exclusion criteria included contraindications to anesthetized endoscopy or a non-HNSCC histopathology. The Ethics Committee of the Medical University of Innsbruck approved this study (Approval number: 1199/2019), and all participants provided written informed consent. Biopsies were obtained from 22 patients with histologically confirmed HNSCC, one sample was tumour free. Tumour tissue from non-necrotic areas was sampled using biopsy forceps during endoscopy under anaesthesia, and was then immediately prepared for slice cultures (SCs). Each sample was cut into 250 μm slices using a Compresstome® VF-310-0Z (Precisionary Instruments LLC, Ashland, MA, USA). For control and treatment purposes, slices were collected for culture at different stages of Compresstome slicing. This means that both early and late slices were included in control and immunostimulation-treated wells to balance the spatial bias.

### Culture conditions

Slice cultures (SC) were submerged in a 24-well plate (Corning Incorporated-Life Sciences, Durham, USA) and maintained under different conditions. Each well was filled with 1 mL of serum-free keratinocyte medium (Keratinocyte SFM; #10724-011, Gibco, Grand Island, NY, USA). This medium was supplemented with Gibco Antibiotic-Antimycotic (#15240062, Thermo Fisher Scientific, Rochester, NY, USA) at a 1:100 dilution, yielding final concentrations of 100 µg/mL streptomycin, 250 ng/mL amphotericin B, and 100 units/mL penicillin (15, 26). In T cell-stimulated conditions, stimulation was induced by adding a 30 µL suspension to each ml of culture medium generated from the T Cell Activation/Expansion Kit (Cat. No. 130-091-441, Miltenyi Biotec, Bergisch Gladbach, Germany) [10]. This kit includes anti-biotin MACSiBead particles and biotinylated antibodies against human CD2, CD3, and CD28, simulating antigen-presenting cells. Production of the suspension was carried out according to the manufacturer’s instructions [10]. Immune stimulation was performed on the day the SC was sectioned and the following day, after which the medium was replaced and the culture continued for an additional three days [31]. 

### Immunostaining procedures and image cytometry

After the cultivation period, slice cultures (SCs) were fixed in 4% paraformaldehyde (#FN-10000-4-1, SAV Liquid Production GMBH, Flintsbach am Inn, Germany), and washed with phosphate-buffered saline (PBS; Fresenius Kabi GmbH, Germany) after at least overnight fixation. Fixed SCs were paraffin embedded using the Histos 5 microwave system (Milestone, Sorisole, Italy). Five µm sections were cut with a Microm microtome (Heidelberg, Germany), followed by dewaxing. Hematoxylin-Eosin (HE) staining was performed using Gill´s hematoxylin and eosin according to the manufacturer’s protocol (#1.05174.0500, Merck KGaA, Germany). Following the method described by Fischer et al. [32], cleaved caspase-3 (CC3) immunohistochemical (IHC) staining was performed using the Ventana Discovery Ultra immunostainer (Ventana Roche, USA) and the CC3 antibody (1:400, polyclonal rabbit, #9661, Cell Signaling Technology, USA). For GrB detection, a mouse monoclonal IgG2b antibody (1:200, #3002-MSM4-P1, Invitrogen, Germany) was used, and for SerpinB9 rabbit monoclonal Cell Signaling Technology antibody (1: 400, Cat. Nr. 37908 S) was applied. A Ventana universal secondary antibody (#05268877) enabled the detection of primary antibodies, with the reaction developed using the Ventana DAB Map Detection Kit (#05266360001, Roche Diagnostics, Germany). Hematoxylin (#5277965001, Roche Diagnostics) was used for nuclear counterstaining. The slides were then dehydrated, mounted with Entellan (MERCK, Germany), and dried overnight.

HIF-1α (rabbit monoclonal IgG, Cat. Nr. SAB5600056; 1:500 dilution; Sigma, Darmstadt, Germany) and VEGFA (mouse monoclonal IgG1, Cat. Nr. AB1316; 1:200 dilution, Abcam, Cambridge, UK) were detected in double immunofluorescence (IF) staining using secondary conjugated antibodies (anti-rabbit Alexa Fluor 594 conjugate and anti-mouse IgG1 Alexa Fluor 488 conjugate, both in 1:200: Invitrogen, Darmstadt Germany). Detection of rabbit and mouse antibodies succeeded as described above. For nuclear counterstaining, 4′,6-diamidino-2-phenylindole (DAPI, 1:46,000, Thermo Fisher Scientific, Germany) was applied, and autofluorescence was reduced using the Vector TrueVIEW Autofluorescence Quenching Kit (#VEC-SP-8400, Vector Laboratories, Newark, CA, USA). Slides were mounted with Vectashield Vibrance (Vector Laboratories).

For both IHC and IF image acquisition, a TissueFAXS PLUS system (TissueGnostics GmbH, Vienna, Austria) was used, incorporating a Zeiss Axio Imager Z2 microscope (Jena, Germany) equipped with an apochromat 20x, 0.6 air lens. For brightfield acquisition the colour Pixelink camera PL-D674CU-CYL (Ottawa, Ontario, Canada), for immunofluorescence, the PCO Pixelfly PF-M-QE camera (Excelitas, Kelheim, Germany) with 16-bit gray shades resolution were applied. All available definable tissue regions were acquired from baseline tissue samples and from control and immunostimulatory kit – treated SCs, using the Tissuefaxs 7.1 software (Tissuegnostics). This allowed individual image cytometry analysis of tissue regions. Due to intra-individual heterogeneity, the analysis approached the definable tissue regions. Regions-based data analysis raised the concern of non-independence of samples, that´s why we performed all statistical evaluations by averaging measurements per patient.

IHC staining intensities of definable individual tissue regions were analyzed using HistoQuest 7.1 software (TissueGnostics), and TissueQuest 7.1 was used for IF. Images were imported into HistoQuest or TissueQuest, where areas unsuitable for analysis were excluded, such as necrotic regions. Cells were identified based on hematoxylin or DAPI counterstaining, and IHC or IF signals were mapped to cell nuclei. Cell nuclear-based segmentation was performed in accordance with our previous publications [31, 32]. The mean intensity values reflect colour intensity for IHC products.

Data output from HistoQuest or TissueQuest includes total cell count, sample area and mean deconvoluted IHC marker intensity at an 8-bit level. TissueQuest works with 16-bit grey levels. For baseline samples, the sum of the HIF-1α and VEGFA IF intensities per cell was used. This was due to the heterogeneous (drop-like) distribution of the HIF-1α signal in the cell nucleus, as well as for comparability purposes. The intensity results were gathered and were not subjected to manipulation, meaning they were directly comparable. Sum or mean intensities consider only ‘events’ in the tissue sample that have a detectable cell nucleus, either by haematoxylin or by DAPI. Eventually, immune stimulatory bead-antibody complexes (due to their biotin content) were detected and excluded from any analysis. This was achieved because the bead complexes did not have cell nuclei or provide recognised haematoxylin signals. Additionally, bead clusters could be excluded from the regions of interest manually. Nevertheless, beads clusters were helpful for induction and effects gradients analysis included in this paper. Signals which had no cell nuclear attribution were not considered. The intensities collected from the samples after induction by the immunostimulatory suspension mix were compared with the control samples when evaluating GrB, CC3 and Serpin B9. These stainings were available as a DAB reaction and the raw data were identified as mean intensity. The mean intensity of the immune-stimulated tissue sections was then related to that of the corresponding control sections. These relative mean intensities were recorded based on regions of the tissue sections. An intensity ratio of the stimulated region to the control above 1.25 was considered stimulated or induced. For CC3, the relative mean intensities of tumour cells were considered. The definition of tumour cells was performed using a previously published morphological gating [29, 33]. The attribution of the SerpinB9 signal to tumour cells is described in detail in Additional File 1 – Supplementary Methods. 

### Data analysis

Slug-positivity was considered if a cluster of cell nuclei was observed in the cancer cell nests, or if a diffuse positive reaction was seen [33, 34]. VEGFA and HIF1α sum intensities were collected for all available regions of all patients. The raw data were plotted patient specifically to present the intra-individual variation. Normal VEGFA sum intensities were 2.65 × 10^6^, normal HIF-1α sum intensities were 2.65 × 10^6^ or lower. The input parameters used were the VEGFA and HIF-1α IF levels, the mean baseline SerpinB9 IHC intensity in tumour cells and the negative/positive Slug and HPV categories. GrB IHC mean intensities after immune stimulation were presented in relation to the average control mean intensities from the same patient. CC3 and SerpinB9 IHC mean intensities in tumour cells only (based on morphological gating; see Additional File 1 – Supplementary Methods) after immune stimulation were presented in relation to the average control mean intensity from the same patient. A relative mean intensity of 1.25 or lower for both GrB and CC3 was considered not to be induced. The scaled intensity values in relation to the input parameters were presented as scatter plots. The dots represent the average for all regions of each patient. One to five regions were available for quantitative evaluation per patient. Depending on the quantified IHC or IF signal, the standard error of measurement as a percentage of the mean ranged from 0.05 to 70.78% (Table 1).

**Table 1:** The ranges of available regions per patient and the standard error of measurement as a percentage of the average for all IHC and IF quantifications performed

Analysis	Analysis type	Available regions per patient	Range of SEM related to the average in %
VEGFA sum intensity baseline	IF	1–5	2.45–37.46
HIF-1α sum intensity baseline	IF	1–5	2.78–38.11
SerpinB9 mean intensity baseline	IHC	2–5	2.00-27.47
SerpinB9 mean intensity control, untreated	IHC	2–5	0.29–20.91
SerpinB9 relative intensity immune stimulated	IHC	1–5	1.07–70.89
Granzyme B mean intensity control, untreated	IHC	1–5	0.50-52.12
Granzyme B relative intensity immune stimulated	IHC	1–4	0.21–64.81
CC3 mean intensity control, untreated	IHC	1–5	0.05–21.1
CC3 relative intensity immune stimulated	IHC	1–5	0.45–47.33

The distribution of data populations was tested by D´Agostino & Pearson normality test, and if the data were not normally distributed the differences between the medians were tested by Mann-Whitney-test. In case of normal distribution the Welch´s test was used to compare means. Correlations were calculated using Spearman’s rank correlation coefficient. Independence of ordinal values was tested by Fisher’s exact test.

The related statistical analysis was performed using SPSS Statistics Ver. 29 (IBM, Armonk, NY USA) or GraphPad Prism Ver. 11 (GraphPad Software, San Diego, CA, USA). 

## Results

Tissue samples of 22 HNSCC patients have been used to create slice cultures. One oropharyngeal tissue sample without tumour involvement was included as a tumour-free reference sample. All comparisons to non-malignant tissue in this study refer to this single control. Of the HNSCC samples, 14 were located in the oropharynx, seven in the oral cavity, and one in the hypopharynx. Of the 22 tumours, six were HPV positive and the rest were negative. Patient ages ranged from 36 to 85 years (average 63.1). Four patients were female and 18 were male (Table 2). The tumour-free patient was male. Background information on HPV was obtained from routine pathological diagnostics based on p16 immunohistochemistry (IHC) and HPV polymerase chain reaction (PCR). Slug IHC was evaluated as described in the Materials and Methods section. These molecular biological background markers are also summarized in Table 2.

**Table 2:** Patient information and tumour locations. F: female; M: male. (n. a. means not available)

Patient	Sex	Age	Location primary tumour	T, *N*, M	Grading	Perineural invasion	p16 / HPV status	CD45/Infiltrate	Slug
1	M	75	Oropharynx	T4, N3b; M0	G3	n.a.	positive	scattered	negative
2	M	57	Oropharynx	T2, N0, M0	n.a.	n.a.	positive	immune exluded	negative
3	F	59	Oropharynx	T2, N2b, M0	G2-3	no	negative	infiltrated	negative
4	M	54	Oropharynx	T2, N0, M0	G2	no	negative	immune exluded	positive
5	F	66	Oropharynx	T3, N1, M0	G2-3	no	negative	infiltrated	negative
6	M	74	Oral cavity	T1, N1, M0	G3	no	negative	scattered	negative
7	M	83	Oropharynx	T3, N2b, M0	G2-3	no	negative	infiltrated	positive
8	M	36	Oral cavity	T3, N0, M0	G3	no	negative	scattered	positive
9	M	65	Oropharynx	T4b, N2b, M0	G2-3	n. a.	negative	scattered	positive
10	M	73	Oropharynx	T1, N0, M0	G2-3	no	negative	scattered	positive
11	M	57	Oropharynx	T2, N0, M0	G2-3	no	negative	infiltrated	negative
12	M	85	Oropharynx	T3, N2, M0	G3	n.a.	positive	immune exluded	negative
13	F	51	Oral cavity	T2, N0, M0	G3	no	positive	infiltrated	negative
14	M	59	Oral cavity	T4a, N2a, M0	G3	yes	negative	infiltrated	negative
15	M	64	Oropharynx	T4, N3, M0	G3	yes	positive	immune exluded	negative
16	M	64	Oral cavity	T2, N0, M0	G2	no	negative	scattered	positive
17	M	40	Oropharynx	T4a, N3, M0	G2	no	negative	scattered	positive
18	F	78	Oropharynx	T2, N0, M0	G3	no	negative	scattered	positive
19	M	57	Oral cavity	T1, N0, M0	G2	no	negative	immune desert	positive
20	M	59	Oral cavity	T2, N2, M0	G3	no	negative	scattered	positive
21	M	76	Hypopharynx	T3, N2c, M0	G3	no	negative	immune exluded	negative
22	M	56	Oropharynx	T2, N2, M0	G3	n. a.	positive	infiltrated	negative
23	M	63	Oropharynx	tumourfree	n. a.	no	negative	infiltrated	negative

### Relationship between Slug and HIF-1α

A molecular circuit of mutual regulation between Slug and HIF1-α was expected based on a previous publication [35], moreover, their co-regulation by the p38MAPK pathway was hypothesized in our previous publication [36].

In accordance to previously established IHC evaluation [37] in baseline (not cultured, not treated) tumour tissues, Slug IHC was categorized to negative and positive (Table 2). Moreover, the baseline tumour tissue samples were immunofluorescent (IF) stained using HIF-1α and VEGFA antibodies. HIF-1α and VEGFA sum intensities were quantified as described in the Methods.

Here we investigated the baseline IF sum intensities of HIF1-α and VEGFA, and the mean intensity of SerpinB9 IHC reactions. We expected higher HIF1-α IHC sum intensities in Slug-positive tumours. Our results presented in Additional File 2 - Supplementary Results revealed that in tissue samples of Slug-positive patients the HIF1-α and VEGFA sum intensities were visibly higher, than in the samples of Slug-negative patients, but the difference was not statistically significant (Additional File 2 - Supplementary Results; 1–2.). SerpinB9 showed the opposite tendency, it was clearly lower in tissue samples corresponding to Slug-positive cases than to Slug-negative ones, but still not significantly (Additional File 2 - Supplementary Results; 3.).

As can be seen in Table 2, all HPV-positive cases were Slug-negative. We performed a Fisher's exact test and found that Slug positivity is not independent of HPV status (p = 0.019). Since Slug and HPV positivity are not independent, and the available HPV-positive cases are low in number, we repeated the analyses only in HPV-negative cases. This analysis did not deliver significant differences (Additional File 2 - Supplementary Results; 1-3.). We also performed a univariate analysis to investigate the effect of Slug-positivity on the three baseline intensity values measured. Similar p-values were obtained, which were not statistically significant (data uploaded to Zenodo; doi: https://doi.org/10.5281/zenodo.15630700).

**Fig. 1:**
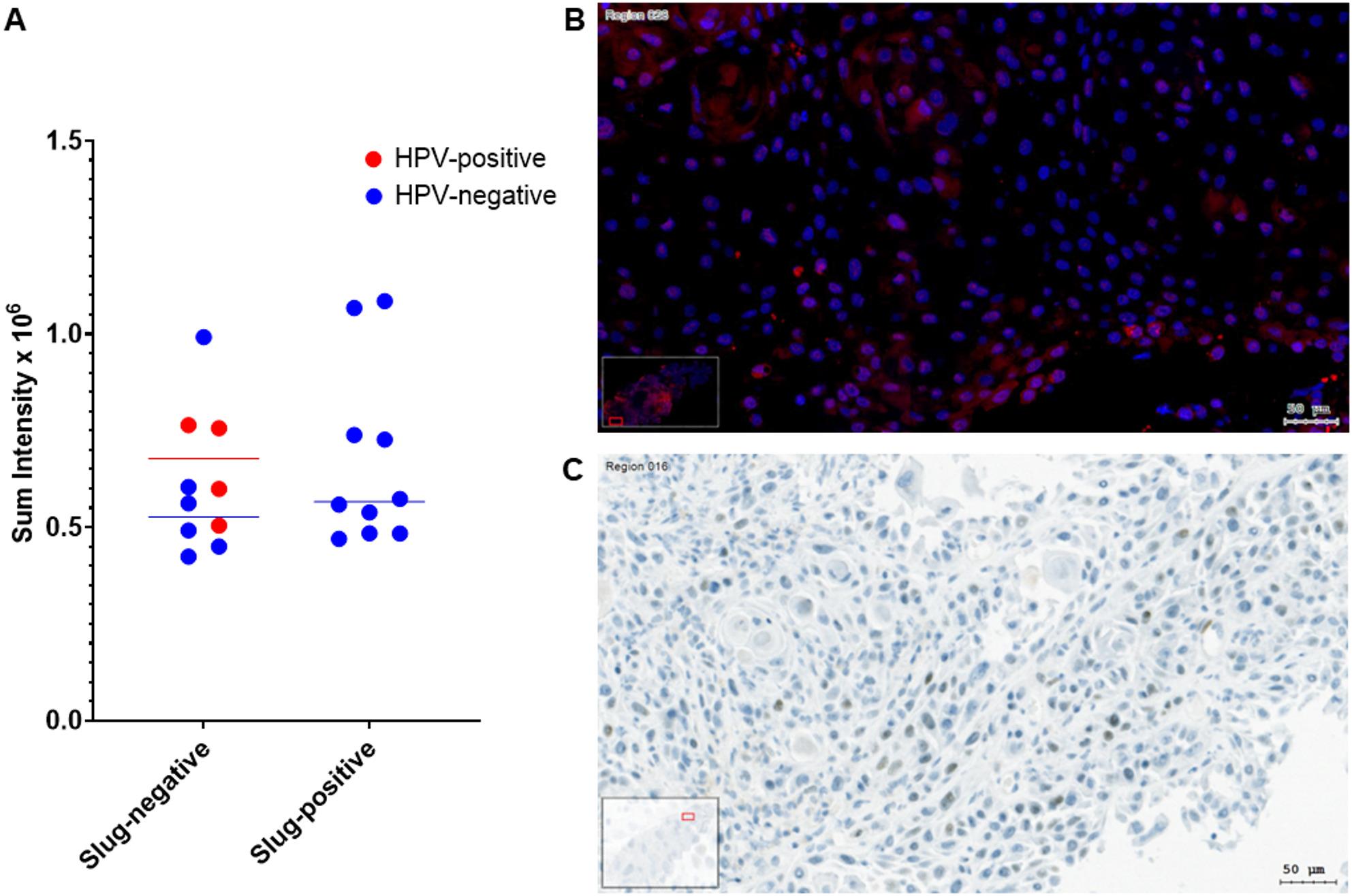
Comparison of sum intensity of HIF-1α in HNSCC tissue samples of Slug- or HPV- positive and negative tumours. **A** Sum IF intensity of HIF-1α in relation to Slug and HPV status. HPV-negative cases are labelled in blue, HPV-positive cases are labelled in red. No significant differences are visible among HPV- or Slug- positive or negative cases in relation to HIF-1α sum intensity. Typical IF for HIF-1α (red) is represented in (**B**), cell nuclei are counterstained with DAPI in blue. Slug IHC with brown cell nuclear detection and blue hematoxylin counterstain is represented in. **C** Heterogeneous distributed red HIF-1α reaction is visible in the nuclei of cells in the cancer cell nest. **B** Similarly to HIF-1α, Slug shows a diffuse reaction in several nuclei of the cancer cell nests. Scale Bar: 50µm on panels (**B**) and (**C**)

In addition to Slug positivity, HPV positivity was investigated to determine whether HIF1-α and VEGFA IHC sum intensities or SerpinB9 mean intensities are influenced by HPV positivity. Similarly to Slug positivity, HPV positivity did not significantly influence these baseline intensity values (data uploaded to Zenodo; DOI: https://doi.org/10.5281/zenodo.15630700). As mentioned before, results indicated that the sum IF intensity of HIF-1α tended to be higher in Slug-positive-tissue than in Slug-negative one, but the difference was not significant (Fig. 1A). Both Slug and HIF-1α were stained positive in the nuclei of tumour cells, indicating a co-regulative relationship (Fig. 1B, C). In Slug-positive HNSCC samples, HIF-1α sum intensities were higher than in Slug-negative samples, although not significantly (Fig. 1A;* n*=10 Slug-positive, 10 Slug negative, *p*=0.53). As previously mentioned, Slug and HPV were not independent of each other, so we analysed the relationship between Slug positivity and HIF-1α sum intensities exclusively in HPV-negative samples. (Fig. 1A, blue labelled dots; *n*=10 Slug-positive, *n*=6 Slug negative, *p*=0.47 with unpaired t-test.). As it is obvious on Fig. 1A, HPV-positive samples did not form a distinguished cluster in terms of HIF-1α sum intensities among slug-negative samples.

Taken together, HIF1-α and VEGFA tended to be increased, and SerpinB9 tended to be decreased in Slug-positive compared to Slug-negative HNSCC tissue. 

**Fig. 2:**
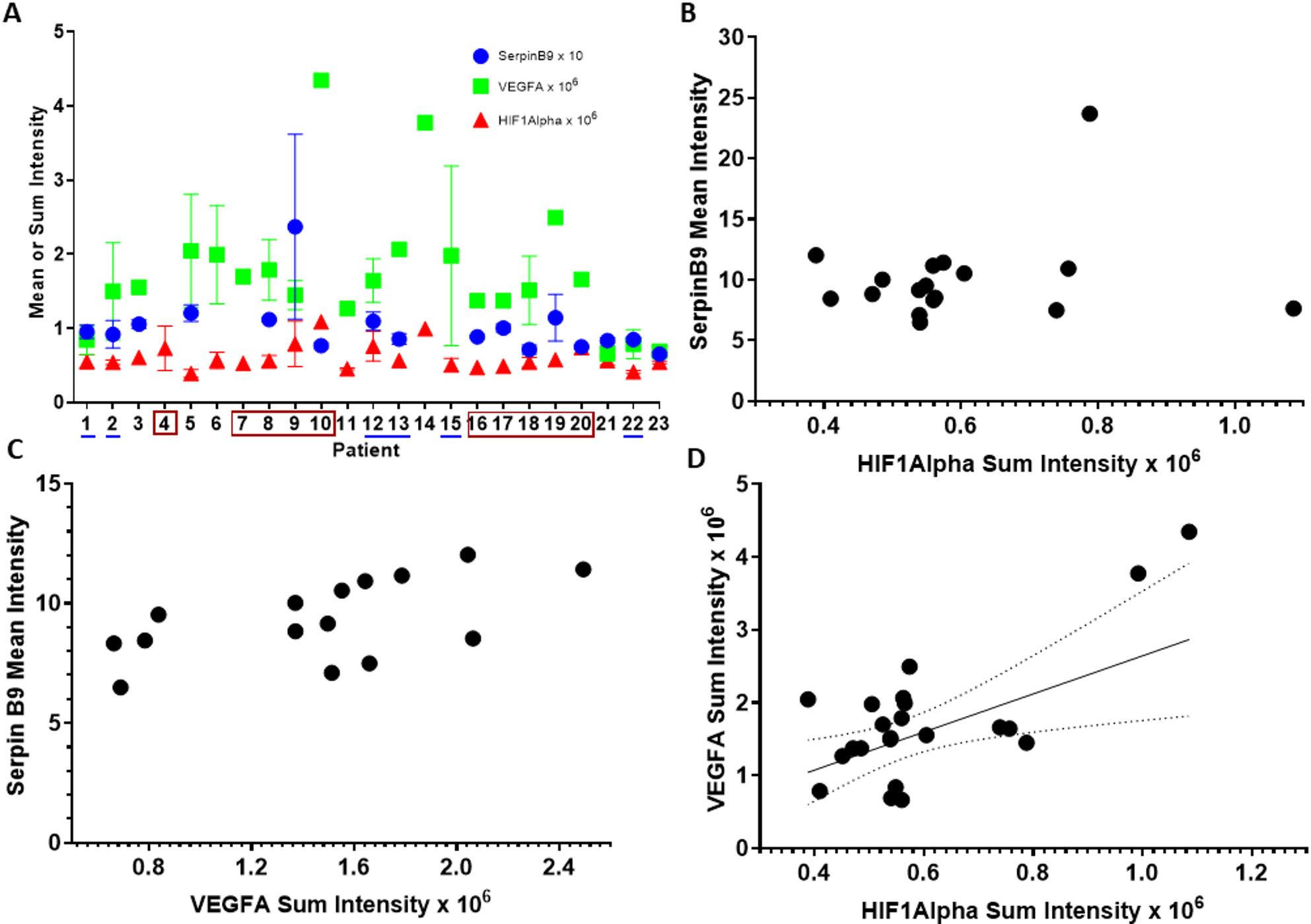
Comparison of baseline sum intensity of HIF1-α, VEGFA and mean intensity of SerpinB9 in the original tissue of 23 patients. Patient 23 was tumour-free. **A** Compared to the single tumour-free tissue sample VEGFA sum intensity was increased in several HNSCC tissue samples, as in those of patients 5-8, 10, 13-15 and 19. HIF1-α sum intensity showed only low baseline levels, similar to the level of the tumour-free patient 23, but it was clearly increased in the tissue of patients 10 and 14. SerpinB9 mean intensity was clearly increased compared to the single tumour-free tissue in tumour samples of patients 5, 8, 9, 12, 19. HPV-positive patient numbers are labelled with blue underlines. Slug-positive patient numbers are labelled with red squares. Correlation was investigated among the intensities of these baseline parameters, no significant correlations were found between HIF1-α and SerpinB9 (p=0.78; **B**) and between VEGFA and SerpinB9 (*p*=0.56; **C**) HIF1-α and VEGFA sum intensities significantly correlated (*p*=0.049; **D**)

The baseline sum intensities of HIF1-α and VEGFA and the mean intensity of SerpinB9 were measured and compared in the original tissue samples of 23 patients, where 22 patients had HNSCC and one patient was tumour-free. VEGFA IF levels were increased in several tumour tissue samples, including some HPV-positive cases (see the blue underlined patient numbers in Fig. 2A) and Slug-positive cases (see the red squared patient numbers in Fig. 2A), compared to the single tumour-free reference sample (23). This was observed in samples of patients 5–8, 10, 13–15, and 19. In the majority of the samples HIF1-α immunofluorescence (IF) sum intensity showed only low baseline levels, similar to those of the single tumour-free patient sample (23). In contrast, HIF1-α IF sum intensity was clearly increased in the tissue of patients 10 and 14, these were HPV-negative. SerpinB9 mean intensity was clearly increased in tumour tissue samples of patients 5, 8, 9, 12, 19, compared to the single tumour-free tissue (23) (Fig. 2A). Patient 12 was HPV-positive. The correlation between the intensities of these baseline parameters was investigated, with no significant correlations found between HIF1-α and SerpinB9 (p = 0.78; see Fig. 2B) or between VEGFA and SerpinB9 (p=0.56; Fig. 2C). HIF1-α and VEGFA IF sum intensities significantly correlated (p=0.049; Fig. 2D). These data fit the knowledge that VEGFA is in major part transcriptionally regulated by HIF1-α [24].

**Fig. 3:**
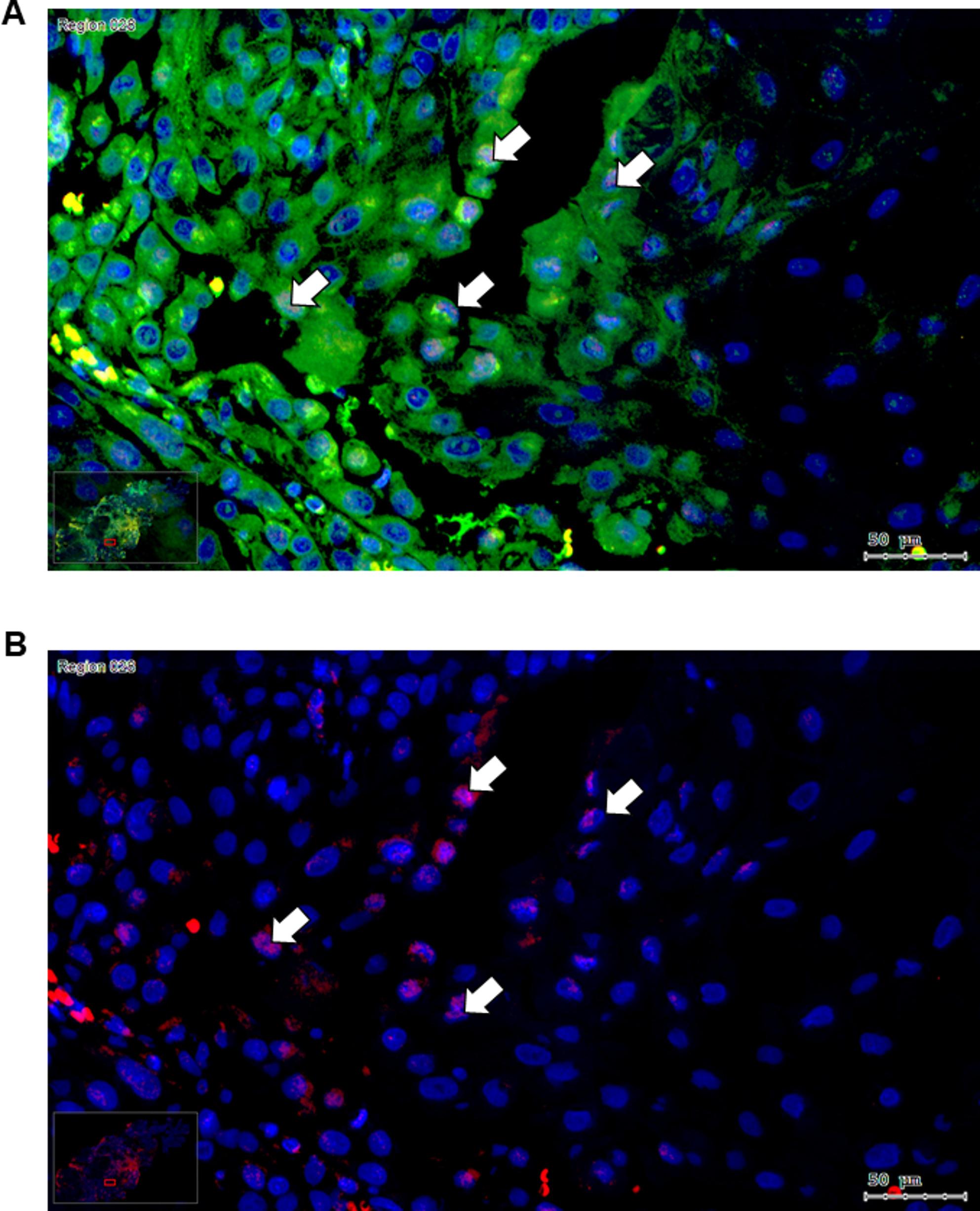
Immunofluorescent staining of VEGFA (green) (**A**, **B**) and HIF-1α (red) (**B**) in tumour tissue sample of patient 10 (**A**: both together; **B**: only HIF-1α). Since the VEGFA IF staining is much stronger, the IF staining of HIF-1α alone is shown separately (**B**). Obvious examples of HIF-1α and VEGFA co-localizations (HIF-1α nuclear and VEGFA cytoplasmatic) are labelled with arrows (Scale Bars: 50µm)

HIF-1α is one of the main regulators of VEGFA. Therefore, the relationship between HIF-1α and VEGFA intensity was investigated in all available tissue samples from 22 HNSCC tumour patients. As shown in Fig. 2D, increased HIF-1α levels were accompanied by increased VEGFA levels. For example, this was true for Patients 10 and 14. VEGFA had higher IF sum intensity than HIF-1α. HIF-1α corresponded more to the cell nuclei of tumour cells in the cancer cell nests, whereas VEGFA was also intense in the stroma, including the endothelial cells. Figure 3 shows tumour-specific colocalization of HIF-1α in the nuclei and VEGFA in the cytoplasm of tumour cells.

### Relationships between induced Granzyme B, increase of apoptosis and SerpinB9

We investigated three outcomes in organotypic slice cultures (SCs) of 23 patients: GrB, CC3 and SerpinB9 mean IHC intensity. These were determined in untreated control and in immune beads stimulated conditions. The immune stimulatory change was calculated related to control mean levels of the samples of the same patient.

We expected stress effects in slice cultures [29] with culture-induced apoptosis, but also GrB and SerpinB9 could have been affected just by the culture of the tissue. As presented in Additional File 2 (Supplementary Results, 4–6), our results demonstrate that the control condition (only SC; untreated) did not significantly influence the outcomes of immune bead stimulation. No significant correlation trends were detectable. Furthermore, it was found that neither HIF-1α nor VEGFA baseline sum intensity levels were significantly correlated with the outcomes of GrB, CC3 or SerpinB9 in control SC conditions (see Additional File 2 – Supplementary Results, Sects. 7–12).

**Fig. 4:**
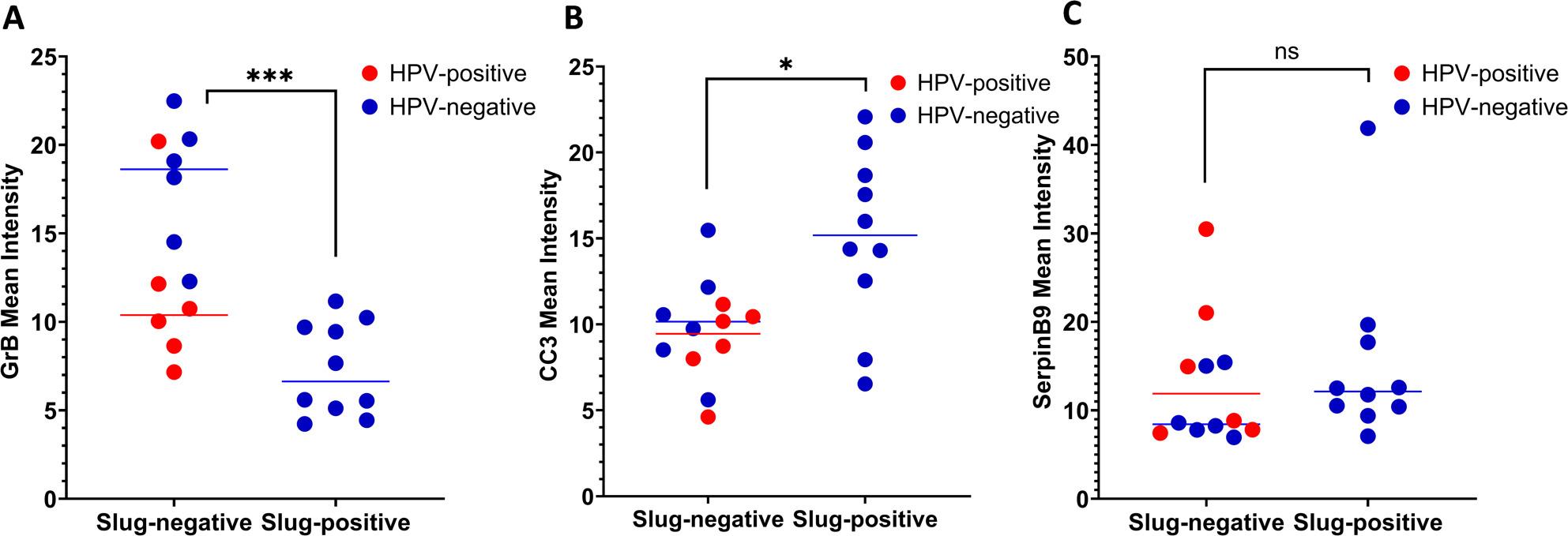
Relationship of GrB (**A**), CC3 (**B**) and SerpinB9 (**C**) mean IHC intensities with the Slug background and HPV-status in untreated SC conditions. In SCs of Slug-positive patients GrB (**A**) was significantly lower (*p*<0.001 by Mann-Whitney test), CC3 (**B**) was significantly higher (*p*=0.01 by Mann-Whitney test) than in SC of Slug-negative patients, whereas SerpinB9 (**C**) was not significantly different (*p*=0.34 by Mann-Whitney test). *N*=12 for Slug-negative patients, and *N*=10 for Slug-positive patients. HPV-negative cases are labelled in blue, HPV-positive cases are labelled in red

Interestingly, the Slug-positive background was significantly related with the GrB and CC3 IHC levels in control SC conditions (Fig. 4). In cultured tissue samples of Slug-positive patients GrB (Fig. 4A) was significantly lower (*p* < 0.001 by Mann-Whitney test), CC3 (Fig. 4B) was significantly higher (*p* = 0.01 by Mann-Whitney test) than in tissue samples of Slug-negative patients, whereas SerpinB9 (Fig. 4C) was not significantly different. Since Slug and HPV are not independent, we repeated the comparison of Slug-negative and positive samples using only HPV-negative tissue samples (blue labelled dots on Fig. 4). GrB levels were significantly lower in Slug-positive cases (*p* = 2 × 10⁻⁴, *N* = 6 for Slug-negative samples and *N* = 10 for Slug-positive tissue samples). CC3 IHC levels did not differ significantly in Slug-positive samples (*p* = 0.072; *N* = 6 for Slug-negative samples and *n* = 10 for Slug-positive tissue samples). The significantly lower CC3 level in Slug-negative tissue samples is more indicative of the effect of HPV-positive tissue samples than of Slug negativity. HPV-positive samples formed a distinct ‘low CC3’ cluster among Slug-negative samples. This can be seen clearly in Fig. 4B. SerpinB9 IHC levels were not significantly different in Slug-positive or negative cultured control tissue samples if only HPV-negative samples were considered (*p* = 0.18 *n* = 6 for slug-negative samples and *n* = 10 for slug-positive tissue samples).

As it was mentioned previously, Slug-positivity was not independent from HPV-status, so we investigated the relationships of GrB, CC3 and SerpinB9 mean IHC intensities in untreated SC conditions with HPV status. GrB and SerpinB9 intensities were not significantly different in untreated tissues of HPV-positive and negative patients (p=0.69 and 0.86 respectively, with Mann-Whitney test), whereas, CC3 intensity was significantly lower in untreated tissues of HPV-positive patients (*p*=0.027 with Mann-Whitney test; Additional File 2 - Supplementary Results; 13. These results indicated that Slug- and HPV-positivity displayed the opposite relationship with CC3 in the untreated, but cultured tissue. 

**Fig. 5:**
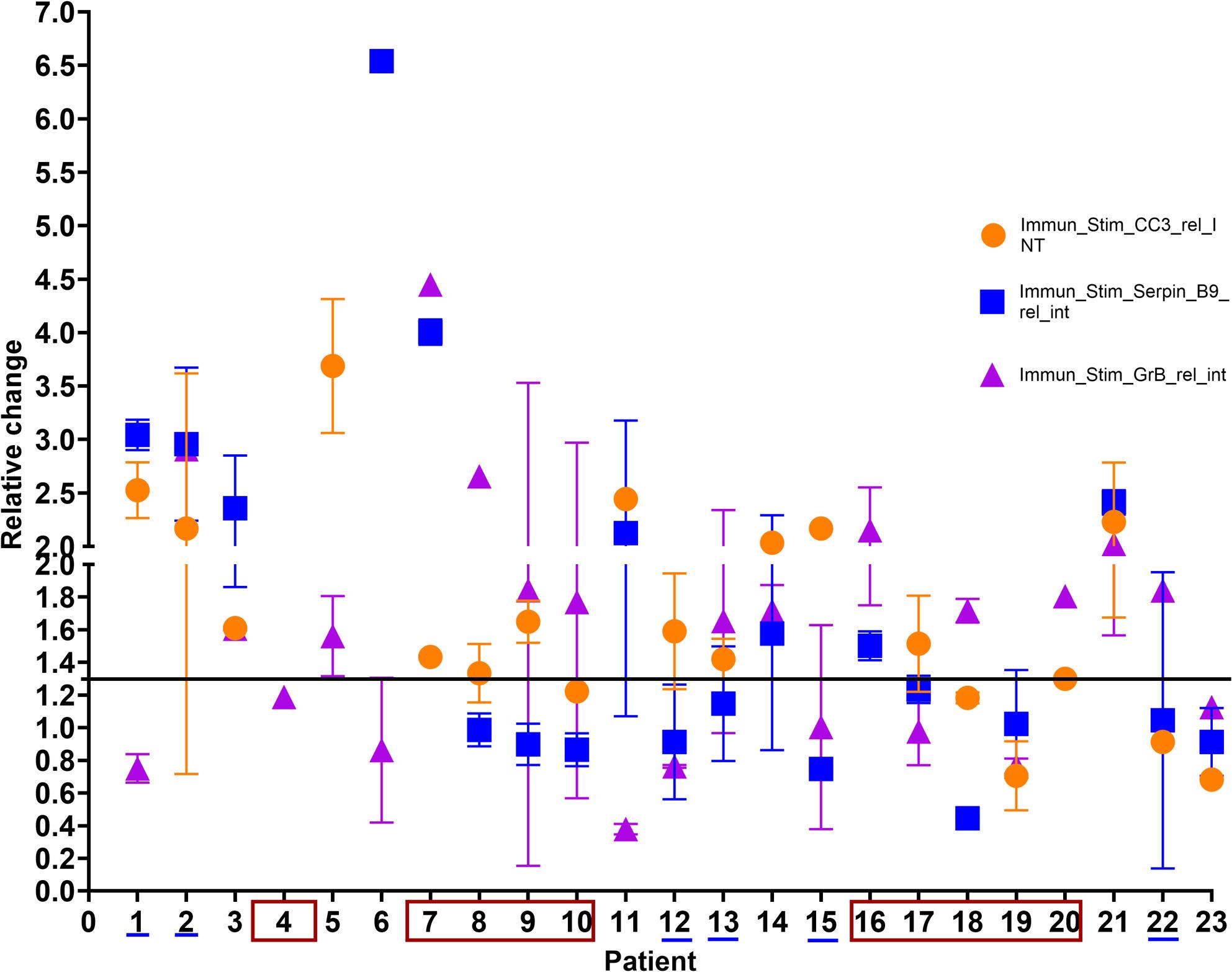
Relative changes of GrB (purple), CC3 (orange) and SerpinB9 (blue) mean IHC intensities after immune stimulatory beads induction. Slug-positive patient numbers are labelled with red squares, HPV-positive patient numbers are labelled with blue underlines of the patient number. Immune stimulation related inductions were considered for values above the line at 1.3 on the Y-axis.

After identifying the effects of slice culture stress, our focus turned to the changes caused by immune stimulation. The mean intensity values in the immune stimulated tissue regions were normalized using the mean intensity values of all the corresponding control regions from the same patient. We investigated changes in the whole tissue in all cells for GrB, and we attributed the changes only to tumour cells as described in Methods and in Additional File 1 - Supplementary Methods, for CC3 and SerpinB9. Changes from 1.25 were considered as ´induction´. GrB was induced after immune stimulation in SCs of patients: 2, 3, 5, 7, 8, 9, 10, 13, 14, 16, 18, 20, 21, 22 (14 of 22 patients sample). In the single tissue sample of the tumour-free patient 23 GrB, CC3 and SerpinB9 were not induced (Fig. 5). CC3 was induced in the tumour cells of patients: 1, 2, 3, 5, 7, 9, 11, 12, 13, 14, 15 and 21 (12 of 19, in samples of 3 patients this value was not available). It is important to mention that CC3 was induced in tissue samples of 5 of 6 HPV-positive patients. Clear and obvious increase was seen in samples of 1, 2, 5, 11, 14, 15, 21 (Fig. 5). None of these were Slug-positive. SerpinB9 was induced in the tumour cells of patient: 1, 2, 3, 6, 7, 11, 14, 16 and 21 (9 of 18, in samples of 4 patients these data were not available). Clear and obvious increase was seen in samples of 1, 2, 3, 7, 11, and 21 (Fig. 5). Patients 1, 2, 12, 13, 15 and 22 are HPV^+^. 

**Fig. 6:**
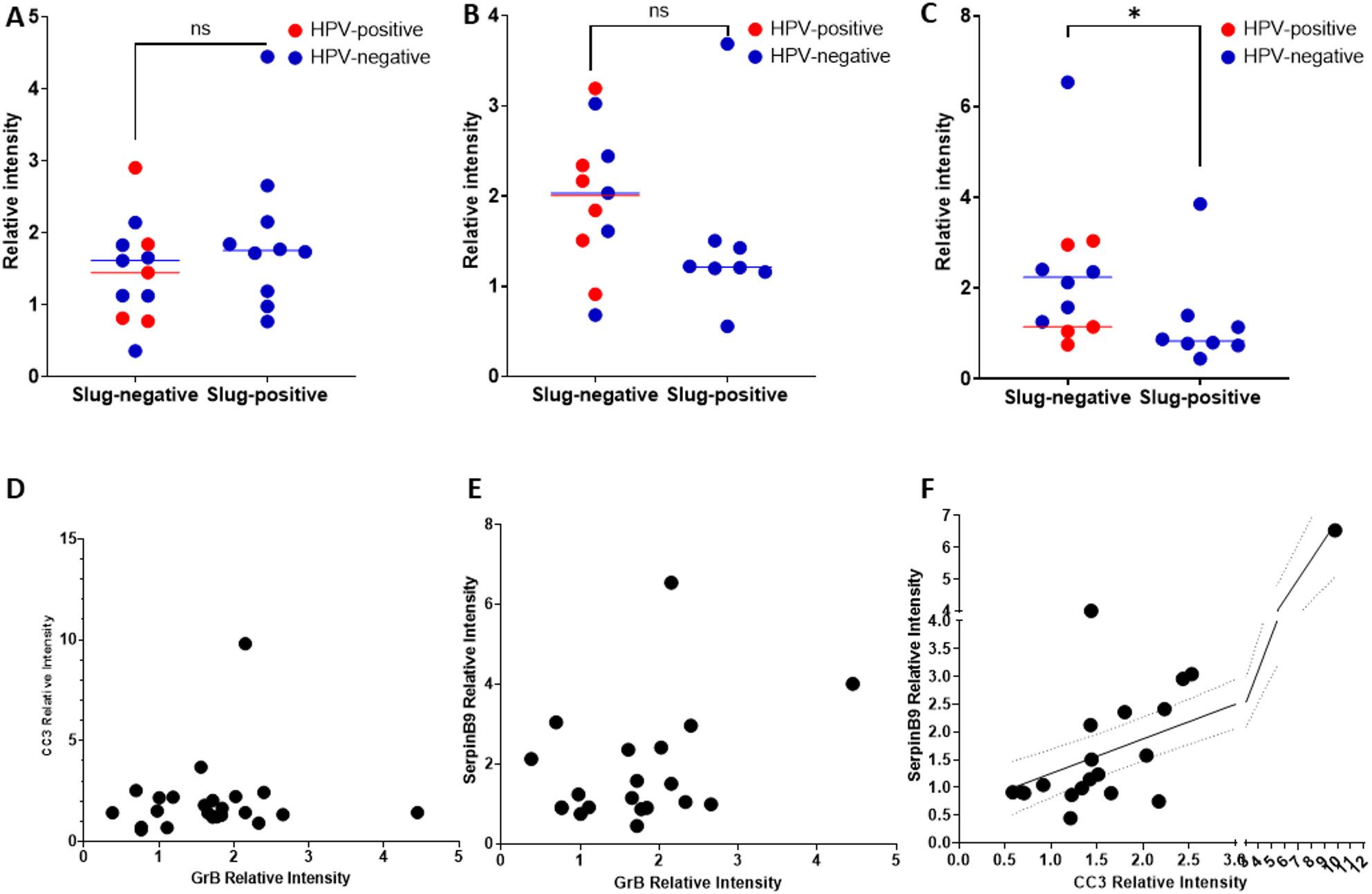
Relative Intensities of GrB (**A**), CC3 (**B**) and SerpinB9 (**C**) after immune stimulation in relation to Slug background. Correlation of Relative Intensities of GrB, CC3 and SerpinB9 with each other (**D**-**F**). **A** GrB relative intensity was not significantly different in Slug negative and positive patients (*p*=0.28 with Mann-Whitney test; *N*=12 vs. 10). **B** CC3 relative intensity was not significantly different in Slug negative and positive patients (*p*=0.09 with Mann-Whitney test; *N*=11 vs. 8). **C** SerpinB9 relative intensity was significantly different in Slug negative and positive patients (p=0.04 with Mann-Whitney test; N=11 vs. 8). A-C: HPV-negative cases are labelled in blue, HPV-positive cases are labelled in red. GrB and CC3 (**D**), GrB and SerpinB9 (**E**) relative intensities did not correlate (*p*=0.66; 0.32), but CC3 and SerpinB9 relative intensities significantly correlated: *p*<0.01; Spearman *r*=0.67 (**F**)

As mentioned above, the presence of Slug-positive tissue background played a role in the relationship between immune stimulation and apoptosis induction. In this context, the relative intensity of GrB, CC3 and SerpinB9 was compared in Slug-negative and Slug-positive patient tissues. (Fig. 6). GrB and CC3 relative intensities were not significantly different in cultured and stimulated tissue samples of Slug-negative and positive patients after immune stimulation (Fig. 6A). If only HPV-negative tissue samples were investigated the differences were also not significant (*p*=0.269 by Mann-Whitney-test for GrB; Slug-negative *n*=7, Slug-positive *n*=10; *p*=0.222 by Mann-Whitney-test for CC3, Slug-negative *n*=5, Slug-positive *n*=8). In contrast, SerpinB9 relative intensities were significantly lower in Slug-positive patient tissues after immune stimulation (Fig. 6C), these were still significantly lower if only HPV-negative tissue samples were considered (*p*=0.02 by Mann-Whitney-test, Slug-negative *n*=6, Slug-positive *n*=8). 

We also investigated if the HPV-positive tissue background played a role in relation of immune stimulation outcomes. None of GrB, CC3 or SerpinB9 relative induced intensities were different in HPV-positive or negative cultured and immune stimulated SCs (p=0.54, 0.64 and 0.98 respectively with Mann-Whitney test). HPV-positive samples did not form distinguished clusters in terms of GrB, CC3 or SerpinB9 relative induced intensities among slug-negative samples (Fig. 6A-C).

The co-regulation potential of the stimulated IHC activities was also investigated. GrB and CC3 (Fig. 6D), as well as GrB and SerpinB9 (Fig. 6E) relative intensities did not show significant correlation, whereas, CC3 and SerpinB9 correlated significantly, with Spearman r=0.67 and p<0.01 (Fig. 6F). These results suggest that, when CC3 is activated by excessive CTL and NK cell activation, tumour cell apoptosis and SerpinB9 cell protection factor activation occurs simultaneously (Fig. 6F).

**Fig. 7:**
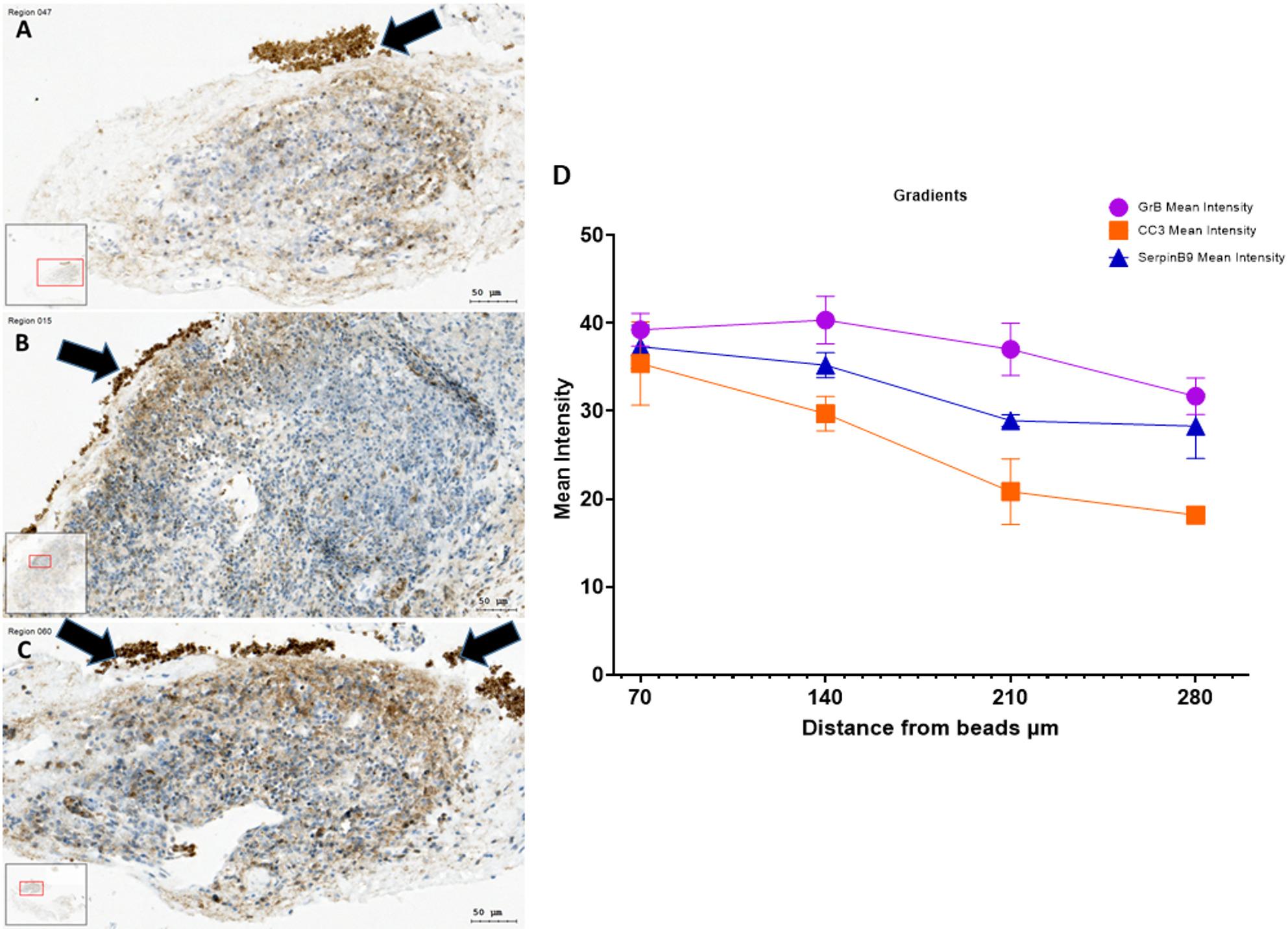
Spatial distribution of GrB (**A**), CC3 (**B**) and SerpinB9 (**C**) signal from the exposition of the immunostimulatory beads. **D** Distance relation of the mean intensity of GrB, CC3 and SerpinB9 from the beads exposure. The Compresstome cut 250 µmeter thick slice cultures were embedded in agarose, or the tissue was covered by agarose, which in some cases trapped and accumulated the immunostimulatory beads (arrows). These beads were still present after fixation, paraffin embedding and microtome sectioning, and were visible after IHC. The beads signals were not included in the intensity quantifications. By increased distance from the beads cluster, the IHC intensity of GrB (**A**, **D**), CC3 (**B**, **D**) and SerpinB9 (**C**, **D**) decreased. The most dramatic decrease was seen for CC3. Scale bars: 50 µm

We would now like to emphasise an important outcome, which indicates that whenever the local immune system was activated to induce cell death in tumour cells, the protective inhibitor SerpinB9, which acts between GrB and the caspase cascade, was also immediately activated. We were able to test this hypothesis within the heterogeneous tumour tissue of some patients, and we now present the spatial analysis of the immune stimulatory beads treated tumor tissue of patient 21. In the cultured and immune stimulated tissue slice of patient 21, all of GrB, CC3 and SerpinB9 were activated (Fig. 5). Figure 7 presents the spatial distribution of all these IHC markers in relation to the immune stimulatory beads treatments. The Compresstome cut 250 µmeter thick slice cultures were embedded in agarose during cutting and culture, or the tissue was covered by agarose, which in some cases trapped and accumulated the immunostimulatory beads (Fig. 7, arrows). These beads were still present after fixation, paraffin embedding and microtome sectioning, and were visible after IHC. The beads signals were excluded from the intensity quantifications in all cases. By increasing distance from the beads cluster, the IHC intensity of GrB (Fig. 7A, D), CC3 (Fig. 7B, D) and SerpinB9 (Fig. 7C, D) decreased. The most dramatic decrease was seen by CC3 (Fig. 7D). These spatial data indicate that higher SerpinB9 than CC3 IHC intensity is maintained in the immune stimulated tumour tissue in tumour cells. This might be interpreted as: tumour cell protection is more intensive than tumour cell death if excessive immune stimulation is achieved in the local tumour immune microenvironment.

## Discussion

This study highlights the complex interplay between immune response, tumour-intrinsic resistance mechanisms, and microenvironmental factors in HNSCC. Using patient-derived tumour SC, we assessed T cell-mediated immune stimulation and its effects on tumour cell apoptosis. In addition, activation of the tumour cell protective SerpinB9, GrB/CC3 inhibitor was investigated. Results indicate that GrB relative IHC intensity was induced in slice cultured and immunostimulatory beads treated tissue of 14 of 22 patients (Fig. 5). High CC3 increase was seen in 7 samples. These data indicate that functional immune activation and the consequent immune-induced tumour cell death is limited in ex vivo cultured and treated HNSCC samples. Both immune-induced GrB and CC3 showed negative tendency to increasing HIF-1α and VEGFA IF sum intensities, but these data did not deliver significant correlations (Additional File 2 -Supplementary Results; 14–15). Further investigative approaches indicated that additional patient-related conditions, as immune infiltrate, exclusion or immune desert might also play a role, the low number of available cases did not allow analysis with further significant outcomes. None of immune-induced GrB, CC3 or SerpinB9 were different in HPV-positive or negative cultured and immune stimulated SC, meaning that also HPV background in this sample contingent did not influence these outcomes.

The immune induction of GrB did not differ between Slug-negative and Slug-positive patients. However, the immune induction of CC3 and SerpinB9 was lower in the tissue samples of Slug-positive patients. In the case of SerpinB9, this difference was significant. For CC3, however, the difference was due to one data point in the Slug-positive dataset being insignificant. These data suggest that immune stimulation-induced tumour cell death and tumour cell protection are low in Slug^+^ patients. Figure 1C shows the localisation of Slug and HIF-1α in the border region of the cancer cell nest. Our previous paper mentioned that these might be regulated together by the p38 MAPK pathway after repeated TGF-β1 treatments [36]. This direction will be further investigated by our research group. HIF-1α and VEGFA IF intensities significantly correlated in the HNSCC tissue samples (Fig. 2D). The IF staining method also revealed co-localisation between HIF-1α and VEGFA in tumour cells of cancer cell nests (Fig. 3). As published studies revealed, inhibition of HIF-1α reduces the levels of HIF-regulated genes, as VEGFA, and EMT-genes as Slug [38, 39].

### Slug positivity and resistance to immune-mediated killing

An important finding of this study was that the majority of tumours with protein stabilized Slug (Slug-positive) were less responsive to immune stimulation (CD3, CD28 activation)-mediated apoptosis. Despite immune stimulation resulting in GrB upregulation in both Slug-positive and Slug-negative tumours, only Slug-negative samples showed a remarkable increase of CC3 (Fig. 5). Among HPV^+^ cases 5 of 6 had increased CC3 after immune stimulation. Slug is known to repress pro-apoptotic genes and E-cadherin while enhancing invasive and immune-evasive phenotypes. In HNSCC, Slug overexpression has been linked to therapy resistance, poor prognosis, and tumour aggressiveness [37]. Therefore, it is likely that Slug interferes with apoptotic pathways downstream of GrB secretion, thereby blocking effective cytotoxicity despite immune cell activity. We have also identified that Slug is not independent of HPV-positivity, Slug-positive cases were all HPV-negative. From the statistical analysis it turned out that none of GrB, CC3 or SerpinB9 relative induced intensities were different in HPV-positive or negative cultured and immune stimulated SCs. Nevertheless, looking at Fig. 5, it is obvious, that in HPV-positive SC samples immune stimulation almost always leads to caspase activation and cell death, and in most cases, it is not accompanied by immunoprotection of the tumor cells. In HPV-negative, slug-negative HNSCC CC3 and SerpinB9 co-stimulation was more frequent. Our results also revealed that Slug is not related with increase of SerpinB9 (Fig. 4C), and it is not the reason of lower induced cell death observed after immune stimulation in HNSCC tumour tissue samples. In Slug-positive tumours both CC3 and SerpinB9 were less activated by immune stimulation. 

### Hypoxia and immunosuppressive signalling via HIF-1α and VEGFA

 Slug protein detection frequently occurred together with nuclear HIF-1α and elevated VEGFA levels, suggesting hypoxia-associated immune resistance [40]. Although, the HIF-1α levels in Slug^+^ samples were not significantly higher, the tendency was visible in Fig. 1A, and the links between EMT and hypoxic signalling are known. HIF-1α stabilizes under hypoxic conditions, promotes VEGFA expression, which in consequence leads to immune suppression and reduced T cell infiltration [24]. In Slug^+^ and HIF-1α-high tumour, as that of Patient 10, CC3 and Serpin B9 activation remained low, despite GrB elevation (Fig. 5), implying that hypoxia-driven pathways may suppress the effectiveness of immune killing. VEGFA, unevenly distributed with enrichment in stromal regions, is known to impair dendritic cell maturation, attract regulatory T cells, and inhibit antigen presentation. All of these changes promote immune exclusion, where immune cells remain in the stroma and fail to infiltrate tumour nests. This immunosuppressive state is further reinforced by Slug and HIF-1α, both of which may dampen apoptotic signalling and CTL function. Therefore, targeting the hypoxia-VEGFA axis, along Slug, could provide a strategy to restore immune responsiveness in resistant tumours.

Our results are in line with findings from other ex vivo and 3D tumour models showing that hypoxia can reduce CC3 activation. For instance, in lung tumour slices, high HIF-1α levels were linked to reduced CC3 staining under low oxygen conditions, suggesting resistance to apoptosis [41]. Similarly, one study found that colon cancer cells under hypoxia had reduced caspase-3 activation after chemotherapy, showing they were more resistant to treatment [42]. Another study by Samuni et al. showed that hypoxia blocked caspase-3 activation caused by radiation in human blood cells [43]. These studies support our finding that hypoxia-related signals may block tumour cell killing, even when immune cells are activated.

### Granzyme B activation

Our results demonstrated that GrB induction was a good indicator of successful immune stimulation and was associated with CC3 increase in numerous patient tumours samples as of patients 2, 5, 14 and 21. However, in only few cases accompanied GrB upregulation apoptosis induction. This reveals that T cell activation, alone is not sufficient for tumour killing, tumour cells apoptosis regulation play important roles. In these cases, apoptotic pathways may appear to be blocked or disrupted, either due to Slug-associated mechanisms or due to other resistance factors like SerpinB9 for example [44]. Interestingly, the higher intensity of CC3 activation came hand in hand with the activation of the apoptosis resistance factor SerpinB9 (Fig. 6F). This highlights the complexity of immune escape mechanisms even in the presence of active cytotoxic T lymphocytes. However, GrB may still serve as a valuable early marker for immune activation, but its interpretation must consider coexisting resistance features.

### SerpinB9 as a limitator of GrB-induced apoptosis

SerpinB9, a protease inhibitor that directly neutralizes GrB, emerged as a potent resistance mechanism in several tumours [16]. In cultured tissue samples of Patient 21, strong SerpinB9 induction accompanied the activation of CC3, which is in agreement with its immune control function [45, 46]. SerpinB9 is not related with EMT, our experimental approaches also did not find any correlation between hypoxia/EMT and SerpinB9. The available literature suggests that it may promote EMT signalling indirectly by supporting tumour cells and carcinoma-associated-fibroblasts (CAFs) persistence [16, 47]. Transcriptomic analysis revealed the relationship between SerpinB9 expression and the upregulation of EMT markers, such as N-cadherin and Vimentin, during tumour progression [48]. SerpinB9 can be further activated under acidic conditions via carbonic anhydrase 9 in the absence of immune stimulation. The upregulation of SerpinB9 in this context may serve to protect tumour cells from apoptosis, thereby supporting tumour growth and survival [49]. In our collective in the tumour tissue of patient 9 elevated level of HIF-1α accompanied high level of SerpinB9. In the majority of the patients tissue samples the SerpinB9 in control (not immune stimulated conditions, was at low level, comparable to the level of a tumour-free tissue (Fig. 2A). In this study SerpinB9 contributed to the secondary, attained immune resistance as consequence of immune activation [50]. SerpinB9 functions as an inhibitor positioned between the activated GrB and the caspase cascade. This hypothesis in context with the corresponding literature indicates that the inhibitor SerpinB9 acts downstream of GrB by blocking caspases. The inhibitory loop of serpins acts as a pseudosubstrate for granzyme B, and forms an irreversible complex with it following cleavage [15]. These findings align with emerging literature positioning SerpinB9 as a key immune escape protein in various cancers, and suggest that its achievement after extensive immune activation may predict resistance to T cell-based therapies [50, 51]. This issue has been clearly demonstrated in our patients-based ex vivo model.

Importantly, these findings have clinical implications. Markers like Slug, HPV-status, HIF-1α, VEGFA, and SerpinB9 could serve as potential biomarkers to predict which patients might benefit from immunotherapy. This could support more personalized treatment decisions. Targeting hypoxia-related pathways may also help to overcome resistance and improve immune cell killing effects. Ex vivo adjustment of the extent of immune activation might help to achieve a balance for tumour cell death instead of tumour cell survival achieved by SerpinB9. A biomarker-guided approach could improve patient selection and support new combination therapies to make immunotherapy more effective in HNSCC. Ex vivo organotypic slice culture treatments help to evaluate the responses of the local immune system to clinical immunotherapies. 

### Limitations

Tumour SCs provide a valuable model to study tumour-immune interactions in a patient-specific context, preserving the architecture and cell diversity of the tumour microenvironment. However, SCs lack systemic factors such as blood circulation and immune cell trafficking, which are important in vivo for full immune and stromal interactions. Therefore, some immune responses observed may not fully represent what happens in the patient.

An important limitation of this study is that comparisons to tumour-free tissue are based on a single control sample. Therefore, these observations should be interpreted as exploratory and do not allow general conclusions regarding differences between tumour and non-malignant tissue.

The use of CD3/CD2/CD28 bead stimulation effectively activates T cells in slice cultures and also induces expression of proteins like SerpinB9, potentially influencing the observed resistance to apoptosis. Further studies using alternative stimulation approaches could clarify this effect. Our analysis focused on apoptosis measured by CC3, but other cell death pathways such as necroptosis, pyroptosis or autophagy were not assessed and may contribute to tumour cell fate. Additionally, we did not evaluate the baseline infiltration or activation status of CD4^+^ and CD8^+^ T cells, which could provide important context on immune function. Tumour extracellular matrix composition and degree of fibrosis or desmoplasia, which can affect immune cell infiltration and function, were also not examined in this study. Due to small tissue size, we were limited in the number and type of immunohistochemical stains and molecular analyses that could be performed. Despite these limitations, SC remains a good model to analyze tumour-immune dynamics in human HNSCC tissue. 

## Conclusions

Our findings demonstrate that while immune stimulation in HNSCC slice cultures can activate immune cells, tumour-intrinsic resistance mechanisms, particularly Slug, HIF-1α and VEGFA might reduce immune activation and effective tumour cell apoptosis. Slug-positive tumours were less responsive to immune-induced tumour cells killing. GrB can indicate early immune activity, but CC3 activation may only occur when further specific conditions, like mitochondrial outer membrane permeabilization, cytochrome c release, and subsequent caspase cascade activation are met. By intensive immune activation in the HNSCC tumour tissue, also the protective inhibitory SerpinB9 was activated. These findings highlight the need for combined therapeutic approaches, tests on patients modelling personalized tissue cultures targeting both the immune system and tumour resistance. At the same time, our organotypic patient-derived model of the local tumour-immune microenvironment allowed a precise preclinical evaluation of the CD2/CD3/CD28 immune stimulation

## Supplementary Information

Supplementary Material 1.

Supplementary Material 2.

## Data Availability

All original raw data, excel tables, Graphpad prism files, SPSS output files and SPSS tables with anonymized data will be loaded to Zenodo at doi: 10.5281/zenodo.15630700 upon acceptance and publication of the paper.

## Abbreviations

CAFs: Carcinoma-associated fibroblasts
CC3: Cleaved caspase 3
CTLs: Cytotoxic T lymphocytes
ECM: Extracellular matrix
EMT: Epithelial mesenchymal transition
GrB: Granzyme B
HIF-1α: Hypoxia-inducible transcription factor 1
HNSCC: Head and neck squamous cell carcinomas
ICI: Checkpoint inhibitors
IF: Immunofluorescence
IHC: Immunohistochemistry
IFN-γ: Interferon-gamma
MHC: Major histocompatibility complex
NK: Natural killer
SC: Slice culture
SCs: Slice cultures
TCR: T cell receptor
TME: Tumour Microenvironment
VEGFA: Vascular endothelial growth factor

## References

[1] Johnson DE, Burtness B, Leemans CR, Lui VWY, Bauman JE, Grandis JR. Head and neck squamous cell carcinoma. Nat reviews Disease primers. 2020;6(1):92. 33243986 10.1038/s41572-020-00224-3 PMC7944998

[2] Curry JM, Sprandio J, Cognetti D, Luginbuhl A, Bar-ad V, Pribitkin E, Tuluc M. Tumor microenvironment in head and neck squamous cell carcinoma. Semin Oncol. 2014;41(2):217–34. 24787294 10.1053/j.seminoncol.2014.03.003

[3] Mamilos A, Lein A, Winter L, Ettl T, Künzel J, Reichert TE, Spanier G, Brochhausen C. Tumor Immune Microenvironment Heterogeneity at the Invasion Front and Tumor Center in Oral Squamous Cell Carcinoma as a Perspective of Managing This Cancer Entity. J Clin Med. 2023;12(4):1704. 10.3390/jcm12041704. 10.3390/jcm12041704 PMC9958892 36836239

[4] Jia Q, Wang A, Yuan Y, Zhu B, Long H. Heterogeneity of the tumor immune microenvironment and its clinical relevance. Experimental Hematol Oncol. 2022;11(1):24. 10.1186/s40164-022-00277-y PMC9034473 35461288

[5] Ryan G. CD3 conformation is crucial for signalling. Nat Rev Immunol. 2010;10(1):7–7.

[6] Ohno H, Ushiyama C, Taniguchi M, Germain RN, Saito T. CD2 can mediate TCR/CD3-independent T cell activation. J Immunol (Baltimore Md: 1950). 1991;146(11):3742–6. 1674520

[7] Esensten JH, Helou YA, Chopra G, Weiss A, Bluestone JA. CD28 Costimulation: From Mechanism to Therapy. Immunity. 2016;44(5):973–88. 27192564 10.1016/j.immuni.2016.04.020 PMC4932896

[8] Garlie NK, LeFever AV, Siebenlist RE, Levine BL, June CH, Lum LG. T cells coactivated with immobilized anti-CD3 and anti-CD28 as potential immunotherapy for cancer. J immunotherapy (Hagerstown Md: 1997). 1999;22(4):336–45. 10.1097/00002371-199907000-00007 10404435

[9] Lum LG, LeFever AV, Treisman JS, Garlie NK, Hanson JP Jr. Immune Modulation in Cancer Patients After Adoptive Transfer of Anti-CD3/Anti-CD28–Costimulated T Cells—Phase I Clinical Trial. J Immunother. 2001;24(5):408–19. 10.1097/00002371-200109000-00003. 10.1097/00002371-200109000-00003 11696696

[10] Green L. Development of an anti-CD2/CD3/CD28 bead-based T-cell proliferation assay. Bioscience Horizons: Int J Student Res. 2014;7:hzu012. 10.1093/biohorizons/hzu012.

[11] Flens MJ, Mulder WM, Bril H, von Blomberg MB, Scheper RJ, van Lier RA. Efficient expansion of tumor-infiltrating lymphocytes from solid tumors by stimulation with combined CD3 and CD28 monoclonal antibodies. Cancer Immunol immunotherapy: CII. 1993;37(5):323–8. 10.1007/BF01518455 PMC11038285 8402736

[12] Waldman AD, Fritz JM, Lenardo MJ. A guide to cancer immunotherapy: from T cell basic science to clinical practice. Nat Rev Immunol. 2020;20(11):651–68. 32433532 10.1038/s41577-020-0306-5 PMC7238960

[13] Raskov H, Orhan A, Christensen JP, Gögenur I. Cytotoxic CD8(+) T cells in cancer and cancer immunotherapy. Br J Cancer. 2021;124(2):359–67. 32929195 10.1038/s41416-020-01048-4 PMC7853123

[14] Liu J, Wang F, Yin D, Zhang H, Feng F. Caspase 3 may participate in the anti-tumor immunity of dendritic cells. Biochem Biophys Res Commun. 2019;511(2):447–53. 30797554 10.1016/j.bbrc.2019.02.081

[15] Trapani JA, Sutton VR. Granzyme B: pro-apoptotic, antiviral and antitumor functions. Curr Opin Immunol. 2003;15(5):533–43. 14499262 10.1016/s0952-7915(03)00107-9

[16] Huang H, Mu Y, Li S. The biological function of Serpinb9 and Serpinb9-based therapy. Front Immunol. 2024;15:1422113. 38966643 10.3389/fimmu.2024.1422113 PMC11222584

[17] Wu L, Seung E, Xu L, Rao E, Lord DM, Wei RR, Cortez-Retamozo V, Ospina B, Posternak V, Ulinski G, et al. Trispecific antibodies enhance the therapeutic efficacy of tumor-directed T cells through T cell receptor co-stimulation. Nat Cancer. 2020;1(1):86–98. 35121834 10.1038/s43018-019-0004-z

[18] Andrews K, Hamers AAJ, Sun X, Neale G, Verbist K, Tedrick P, Nichols KE, Pereira S, Geraghty DE, Pillai AB. Expansion and CD2/CD3/CD28 stimulation enhance Th2 cytokine secretion of human invariant NKT cells with retained anti-tumor cytotoxicity. Cytotherapy. 2020;22(5):276–90. 32238299 10.1016/j.jcyt.2020.01.011 PMC7666372

[19] Fu Z, Mowday AM, Smaill JB, Hermans IF, Patterson AV. Tumour Hypoxia-Mediated Immunosuppression: Mechanisms and Therapeutic Approaches to Improve Cancer Immunotherapy. Cells. 2021;10(5):1006. 10.3390/cells10051006. 10.3390/cells10051006 PMC8146304 33923305

[20] Arner EN, Rathmell JC. Metabolic programming and immune suppression in the tumor microenvironment. Cancer Cell. 2023;41(3):421–33. 36801000 10.1016/j.ccell.2023.01.009 PMC10023409

[21] Wu Q, You L, Nepovimova E, Heger Z, Wu W, Kuca K, Adam V. Hypoxia-inducible factors: master regulators of hypoxic tumor immune escape. J Hematol Oncol. 2022;15(1):77. 35659268 10.1186/s13045-022-01292-6 PMC9166526

[22] Hu J, Li X, Yang L, Li H. Hypoxia, a key factor in the immune microenvironment. Biomed Pharmacother. 2022;151:113068. 35676780 10.1016/j.biopha.2022.113068

[23] Geindreau M, Ghiringhelli F, Bruchard M. Vascular Endothelial Growth Factor, a Key Modulator of the Anti-Tumor Immune Response. Int J Mol Sci. 2021;22(9):4871. 10.3390/ijms22094871. 10.3390/ijms22094871 PMC8124522 34064508

[24] Ahluwalia A, Tarnawski AS. Critical role of hypoxia sensor–HIF-1α in VEGF gene activation. Implications for angiogenesis and tissue injury healing. Curr Med Chem. 2012;19(1):90–7. 22300081 10.2174/092986712803413944

[25] Ghosh R, Samanta P, Sarkar R, Biswas S, Saha P, Hajra S, Bhowmik A. Targeting HIF-1α by Natural and Synthetic Compounds: A Promising Approach for Anti-Cancer Therapeutics Development. Molecules. 2022;27(16):5192. 10.3390/molecules27165192. 10.3390/molecules27165192 PMC9413992 36014432

[26] Luo H, Vong CT, Chen H, Gao Y, Lyu P, Qiu L, Zhao M, Liu Q, Cheng Z, Zou J, et al. Naturally occurring anti-cancer compounds: shining from Chinese herbal medicine. Chin Med. 2019;14:48. 31719837 10.1186/s13020-019-0270-9 PMC6836491

[27] Elmusrati A, Wang J, Wang C-Y. Tumor microenvironment and immune evasion in head and neck squamous cell carcinoma. Int J Oral Sci. 2021;13(1):24. 34341329 10.1038/s41368-021-00131-7 PMC8329257

[28] Carter EP, Roozitalab R, Gibson SV, Grose RP. Tumour microenvironment 3D-modelling: simplicity to complexity and back again. Trends cancer. 2021;7(11):1033–46. 34312120 10.1016/j.trecan.2021.06.009

[29] Greier MDC, Runge A, Dudas J, Carpentari L, Schartinger VH, Randhawa A, Mayr M, Petersson M, Riechelmann H. Optimizing culturing conditions in patient derived 3D primary slice cultures of head and neck cancer. Front Oncol. 2023;13:1145817. 37064104 10.3389/fonc.2023.1145817 PMC10101142

[30] Runge A, Mayr M, Schwaiger T, Sprung S, Chetta P, Gottfried T, Dudas J, Greier MC, Glatz MC, Haybaeck J, et al. Patient-derived head and neck tumor slice cultures: a versatile tool to study oncolytic virus action. Sci Rep. 2022;12(1):15334. 36097280 10.1038/s41598-022-19555-0 PMC9467994

[31] Greier MDC, Runge A, Dudas J, Hartl R, Santer M, Dejaco D, Steinbichler TB, Federspiel J, Seifarth C, Konschake M, et al. Cytotoxic response of tumor-infiltrating lymphocytes of head and neck cancer slice cultures under mitochondrial dysfunction. Front Oncol. 2024;14:1364577. 38515569 10.3389/fonc.2024.1364577 PMC10954813

[32] Giotakis AI, Dudas J, Glueckert R, Dejaco D, Ingruber J, Fleischer F, Innerhofer V, Pinggera L, Bektic-Tadic L, Gabriel SAM, et al. Characterization of epithelial cells, connective tissue cells and immune cells in human upper airway mucosa by immunofluorescence multichannel image cytometry: a pilot study. Histochem Cell Biol. 2021;155(3):405–21. 33251550 10.1007/s00418-020-01945-y PMC8021535

[33] Riechelmann H, Steinbichler TB, Sprung S, Santer M, Runge A, Ganswindt U, Gamerith G, Dudas J. The Epithelial-Mesenchymal Transcription Factor Slug Predicts Survival Benefit of Up-Front Surgery in Head and Neck Cancer. Cancers. 2021;13(4):772. 10.3390/cancers13040772. 10.3390/cancers13040772 PMC7918715 33673269

[34] Federspiel J, Steinbichler TB, Vorbach SM, Eling MT, Borena W, Seifarth C, Hofauer BG, Dudas J. Patient-Derived Cancer-Associated Fibroblasts Support the Colonization of Tumor Cells in Head and Neck Squamous Cell Carcinoma. Biomedicines. 2025;1313(2):358. 10.3390/biomedicines13020358. 10.3390/biomedicines13020358 PMC11852712 40002772

[35] Penolazzi L, Bonaccorsi G, Gafa R, Ravaioli N, Gabriele D, Bosi C, Lanza G, Greco P, Piva R. SLUG/HIF1-alpha/miR-221 regulatory circuit in endometrial cancer. Gene. 2019;711:143938. 31220580 10.1016/j.gene.2019.06.028

[36] Federspiel J, Greier MDC, Ladanyi A, Dudas J. p38 Mitogen-Activated Protein Kinase Inhibition of Mesenchymal Transdifferentiated Tumor Cells in Head and Neck Squamous Cell Carcinoma. Biomedicines. 2023;11(12):3301. 10.3390/biomedicines11123301. 10.3390/biomedicines11123301 PMC10741606 38137525

[37] Steinbichler TB, Dudas J, Ingruber J, Glueckert R, Sprung S, Fleischer F, Cidlinsky N, Dejaco D, Kofler B, Giotakis AI, et al. Slug Is A Surrogate Marker of Epithelial to Mesenchymal Transition (EMT) in Head and Neck Cancer. J Clin Med. 2020;9(7):2061. 10.3390/jcm9072061. 10.3390/jcm9072061 PMC7408865 32630033

[38] Wei Z, Shan Y, Tao L, Liu Y, Zhu Z, Liu Z, Wu Y, Chen W, Wang A, Lu Y. Diallyl trisulfides, a natural histone deacetylase inhibitor, attenuate HIF-1alpha synthesis, and decreases breast cancer metastasis. Mol Carcinog. 2017;56(10):2317–31. 28574600 10.1002/mc.22686

[39] Kim JH, Hwang YJ, Han SH, Lee YE, Kim S, Kim YJ, Cho JH, Kwon KA, Kim JH, Kim SH. Dexamethasone inhibits hypoxia-induced epithelial-mesenchymal transition in colon cancer. World J Gastroenterol. 2015;21(34):9887–99. 26379394 10.3748/wjg.v21.i34.9887 PMC4566382

[40] Wiechec E, Matic N, Ali A, Roberg K. Hypoxia induces radioresistance, epithelial–mesenchymal transition, cancer stem cell–like phenotype and changes in genes possessing multiple biological functions in head and neck squamous cell carcinoma. Oncol Rep. 2022;47(3):58. 35059742 10.3892/or.2022.8269 PMC8808704

[41] Dong M, Böpple K, Thiel J, Winkler B, Liang C, Schueler J, Davies EJ, Barry ST, Metsalu T, Mürdter TE, et al. Perfusion Air Culture of Precision-Cut Tumor Slices: An Ex Vivo System to Evaluate Individual Drug Response under Controlled Culture Conditions. Cells. 2023;12(5):807. 10.3390/cells12050807. 10.3390/cells12050807 PMC10001200 36899943

[42] Mayes PA, Dolloff NG, Daniel CJ, Liu JJ, Hart LS, Kuribayashi K, Allen JE, Jee DI, Dorsey JF, Liu YY, et al. Overcoming hypoxia-induced apoptotic resistance through combinatorial inhibition of GSK-3β and CDK1. Cancer Res. 2011;71(15):5265–75. 21646472 10.1158/0008-5472.CAN-11-1383 PMC3667402

[43] Samuni AM, Kasid U, Chuang EY, Suy S, Degraff W, Krishna MC, Russo A, Mitchell JB. Effects of hypoxia on radiation-responsive stress-activated protein kinase, p53, and caspase 3 signals in TK6 human lymphoblastoid cells. Cancer Res. 2005;65(2):579–86. 15695402

[44] Chowdhury D, Lieberman J. Death by a thousand cuts: granzyme pathways of programmed cell death. Annu Rev Immunol. 2008;26:389–420. 18304003 10.1146/annurev.immunol.26.021607.090404 PMC2790083

[45] Han R, Yu L, Zhao C, Li Y, Ma Y, Zhai Y, Qian Z, Gu Y, Li S. Inhibition of SerpinB9 to enhance granzyme B-based tumor therapy by using a modified biomimetic nanoplatform with a cascade strategy. Biomaterials. 2022;288:121723. 35963816 10.1016/j.biomaterials.2022.121723

[46] Bird CH, Christensen ME, Mangan MSJ, Prakash MD, Sedelies KA, Smyth MJ, Harper I, Waterhouse NJ, Bird PI. The granzyme B-Serpinb9 axis controls the fate of lymphocytes after lysosomal stress. Cell Death Differ. 2014;21(6):876–87. 24488096 10.1038/cdd.2014.7 PMC4013521

[47] Tuomela K, Ambrose AR, Davis DM. Escaping Death: How Cancer Cells and Infected Cells Resist Cell-Mediated Cytotoxicity. Front Immunol. 2022;13:867098. 35401556 10.3389/fimmu.2022.867098 PMC8984481

[48] Ibáñez-Molero S, Alex vV, Joanna P, Karlijn H, Kim MTA, Joyce M, Ingrid S, Ewout H, Yannick L. SERPINB9 is commonly amplified and high expression in cancer cells correlates with poor immune checkpoint blockade response. OncoImmunology. 2022;11(1):2139074. 36465485 10.1080/2162402X.2022.2139074 PMC9710519

[49] Harada M, Kotani H, Iida Y, Tanino R, Minami T, Komohara Y, Yoshikawa K, Uemura H. Hypoxia-related carbonic anhydrase 9 induces serpinB9 expression in cancer cells and apoptosis in T cells via acidosis. Cancer Sci. 2024;115(5):1405–16. 38413363 10.1111/cas.16133 PMC11093193

[50] Cunningham TD, Jiang X, Shapiro DJ. Expression of high levels of human proteinase inhibitor 9 blocks both perforin/granzyme and Fas/Fas ligand-mediated cytotoxicity. Cell Immunol. 2007;245(1):32–41. 17490628 10.1016/j.cellimm.2007.03.004 PMC3655900

[51] Ibáñez-Molero S, van Vliet A, Pozniak J, Hummelink K, Terry AM, Monkhorst K, Sanders J, Hofland I, Landeloos E, Van Herck Y, et al. SERPINB9 is commonly amplified and high expression in cancer cells correlates with poor immune checkpoint blockade response. Oncoimmunology. 2022;11(1):2139074. 36465485 10.1080/2162402X.2022.2139074 PMC9710519

