# Supplementary material for: Granzyme B, HIF-1α, VEGFA and SerpinB9 in the local tumour immune system of immune-stimulated slice cultures of head and neck cancer
Source: BMC Cancer. 2026 Jun 11;26:992. doi: 10.1186/s12885-026-16282-x (PMC13483648; doi:10.1186/s12885-026-16282-x)
Supplement: Supplementary file 1 [file 12885_2026_16282_MOESM1_ESM.docx]

**Supplementary Methods**

**Image cytometry analyses**

Image cytometry was performed using HistoQuest (TissueFAXS), which was implemented to standardize quantitative signal detection from stained tissue sections. For each sample, 3–5 regions were analyzed.

Before segmentation, markers were manually assigned in the software: haematoxylin (H) as the “*master”* channel and SerpinB9 or other experimental markers as the “*original”* channel. The morphological parameters selected for quantitative analysis included: area, mean intensity, equivalent diameter, perimeter, compactness, and eccentricity. After confirming the correct colour classification for each marker, a freehand ROI was drawn within one representative region that contained both tumor and non-tumoral tissue. To ensure that the downstream quantification selectively reflected authentic cancer cells, segmentation parameters were optimized based on the morphological properties of the distinct cell types present.

Initially, nuclear segmentation was defined on the H channel, with the goal of labelling nuclei accurately while avoiding fragmentation or oversizing. Final settings included a nuclear size threshold of 10 and a background threshold of 6 (adjusted according to grayscale values). Once the software reliably recognized nuclei across all regions, cellular segmentation was established on the experimental marker channel. Multiple trials were performed using the ring mask function; however, the optimal settings for this study were achieved without using the ring mask, and instead by applying the identified cell mask (inside and outside), with a maximum growing step of 1.1 μm and 0 μm skip steps. Moreover, a nuclei mask was also applied, with the background threshold manually checked against grayscale intensities and set to 9. All segmentation parameters were validated across the 3–5 regions prior to quantitative measurement.

To train the software to distinguish clusters of tumor cells specifically, a second freehand ROI was drawn containing mixed malignant and healthy tissue. Scatter diagrams were generated by plotting the area (x-axis) against each morphological parameter (y-axis): equivalent diameter, perimeter, compactness, and eccentricity. Diagrams without clear population separation were excluded. The area–eccentricity plot offered the best discrimination, allowing the identification of a distinct tumor-cell cluster using two cut-offs: eccentricity ≥ 0.9 and area ≥ 42 µm² (bottom-right cluster). These settings effectively excluded non-tumoral cells such as fibroblasts, stromal cells, and endothelial cells.

Finally, to maximize the accuracy of tumor-specific quantification, freehand ROIs were generated within sub-regions highly enriched in tumor cells and cancer cell nests, intentionally excluding surrounding non-tumoral tissue. Within these tumor-dense regions, the mean intensity of SerpinB9 and the total number of cells were measured. All described settings were applied consistently across all samples included in the study, with no outliers detected.
