# Supplementary material for: Granzyme B, HIF-1α, VEGFA and SerpinB9 in the local tumour immune system of immune-stimulated slice cultures of head and neck cancer
Source: BMC Cancer. 2026 Jun 11;26:992. doi: 10.1186/s12885-026-16282-x (PMC13483648; doi:10.1186/s12885-026-16282-x)
Supplement: Supplementary file 2 [file 12885_2026_16282_MOESM2_ESM.docx]

**Supplementary results.**

1. Not Significant difference of HIF1-α baseline sum intensity related to Slug-positivity.

Mann-Whitney test, p=0.55.

Slug and HPV are not independent, so we repeated the analysis only in HPV-negative cases. The difference in Slug-positive and negative cases was not significant; p=0.758 with Mann-Whitney test.

2. Not Significant difference of VEGFA baseline sum intensity related to Slug-positivity.

Mann-Whitney U test, p=0.47.

Slug and HPV are not independent, so we repeated the analysis only in HPV-negative cases. The difference in Slug-positive and negative cases was not significant; p=0.606 with Mann-Whitney test.

3. Not Significant difference of SerpinB9 baseline mean intensity related to Slug.

Welch´s test p=0.062.

Slug and HPV are not independent, so we repeated the analysis only in HPV-negative cases. The difference in Slug-positive and negative cases was not significant; p=0.106 with Welch´s t-test test.

4. Not significant correlation between GrB control slice culture IHC mean intensity levels and immunostimulated levels in available regions of all patients.

In 23 patients derived tissue samples the mean GrB IHC intensity in control slice cultured (N=55) and in immunostimulatory beads treated conditions (N=51) are presented by the graph. The IHC levels were compared in a patients – based way. The standard errors of measurements error bars represent the standard errors for both X and Y axis values. The mean intensities do not correlate significantly, p=0.08. The linear regression trend line is presented in blue.

5. Not significant correlation between cleaved caspase 3 (CC3) control slice culture IHC mean intensity levels and immunostimulated levels in available regions of all patients.

In 23 patients derived tissue samples the mean CC3 IHC intensity in control slice cultured (N=43) and in immunostimulatory beads treated conditions (N=41) are presented by the graph. The IHC levels were compared in a patients – based way. The standard errors of measurements error bars represent the standard errors for both X and Y axis values. The mean intensities do not correlate significantly, p=0.064. The linear regression trend line is presented in blue.

6. Not significant correlation between SerpinB9 control slice culture IHC mean intensity levels and immunostimulated levels in available regions of all patients.

In 23 patients derived tissue samples the mean SerpinB9 IHC intensity in control slice cultured (N=64) and in immunostimulatory beads treated conditions (N=77) are presented by the graph. The IHC levels were compared in a patients – based way. The standard errors of measurements error bars (SEM) represent the standard errors for both X and Y axis values. The mean intensities do not correlate significantly, p=0.58. The linear regression trend line is presented in blue.

7. Not significant correlation between GrB control slice culture IHC mean intensity levels and baseline HIF-1α sum intensity .

In 23 patients derived tissue samples the mean GrB IHC intensity in control slice cultured (N=55) conditions were compared with the mean ± SEM of HIF1-α baseline sum intensity in a patients – based way. The standard errors of measurements error bars represent the standard errors for both -X and Y-axis values. The X and Y values do not correlate significantly, p=0.92. The linear regression trend line is presented in blue.

8. Not significant correlation between GrB control slice culture IHC mean intensity levels and baseline VEGFA sum intensity .

In 23 patients derived tissue samples the mean GrB IHC intensity in control slice cultured (N=55) conditions were compared with the mean ± SEM of VEGFA baseline sum intensity in a patients – based way. The standard errors of measurements error bars represent the standard errors for both -X and Y-axis values. The X and Y values do not correlate significantly, p=0.46. The linear regression trend line is presented in blue.

9. Not significant correlation between CC3 control slice culture IHC mean intensity levels and baseline HIF-1α sum intensity .

In 23 patients derived tissue samples the mean CC3 IHC intensity in control slice cultured (N=43) conditions were compared with the mean ± SEM of baseline HIF1-α sum intensity in a patients – based way. The standard errors of measurements error bars represent the standard errors for both -X and Y-axis values. The X and Y values do not correlate significantly, p=0.63. The linear regression trend line is presented in blue.

10. Not significant correlation between CC3 control slice culture IHC mean intensity levels and baseline VEGFA sum intensity .

In 23 patients derived tissue samples the mean CC3 IHC intensity in control slice cultured (N=43) conditions were compared with the mean ± SEM of baseline VEGFA sum intensity in a patients – based way. The standard errors of measurements error bars represent the standard errors for both -X and Y-axis values. The X and Y values do not correlate significantly, p=0.46. The linear regression trend line is presented in blue.

11. Not significant correlation between SerpinB9 control slice culture IHC mean intensity levels and baseline HIF-1α sum intensity .

In 23 patients derived tissue samples the mean CC3 IHC intensity in control slice cultured (N=64) conditions were compared with the mean ± SEM of baseline HIF1-α sum intensity in a patients – based way. The standard errors of measurements error bars represent the standard errors for both -X and Y-axis values. The X and Y values do not correlate significantly, p=0.96. The linear regression trend line is presented in blue.

12. Not significant correlation between SerpinB9 control slice culture IHC mean intensity levels and baseline VEGFA sum intensity .

In 23 patients derived tissue samples the mean SerpinB9 IHC intensity in control slice cultured (N=64) conditions were compared with the mean ± SEM of baseline VEGFA sum intensity in a patients – based way. The standard errors of measurements error bars represent the standard errors for both -X and Y-axis values. The X and Y values do not correlate significantly, p=0.46. The linear regression trend line is presented in blue.

13. Relationship of CC3 IHC intensities with the HPV background in untreated SC conditions.

In cultured tissue samples of HPV-positive patients (n=16) CC3 was significantly lower (p=0.027 with Mann-Whitney test) than in HPV-negative patients (n=6).

14. Not significant tendential negative relationship between increasing VEGFA sum IF intensity and immune stimulation induced changes of GrB, CC3 and SerpinB9.

|  | VEGFA  sum intensity x 10^6^ vs. Immun_Stim_GrB_rel_int | VEGFA  sum intensity x 10^6^ vs. Immun_Stim_CC3_rel_INT | VEGFA  sum intensity x 10^6^ vs. Immun_Stim_Serpin_B9_rel_int |
| --- | --- | --- | --- |
| Spearman r |  |  |  |
| r | -0,03783 | -0,1173 | -0,1684 |
| 95% confidence interval | -0,4627 to 0,4012 | -0,5422 to 0,3554 | -0,5780 to 0,3089 |
|  |  |  |  |
| P value |  |  |  |
| P (two-tailed) | 0,8673 | 0,6224 | 0,4778 |
| P value summary | ns | ns | ns |
| Exact or approximate P value? | Approximate | Approximate | Approximate |
| Significant? (alpha = 0.05) | No | No | No |
|  |  |  |  |
| Number of XY Pairs | 22 | 20 | 20 |

15. Not significant tendential negative relationship between increasing HIF-1α sum IF intensity and immune stimulation induced changes of GrB, CC3 and SerpinB9.

|  | HIF1  sum intensity x 10^6^ vs. Immun_Stim_GrB_rel_int | HIF1  sum intensity x 10^6^ vs. Immun_Stim_CC3_rel_INT | HIF1  sum intensity x 10^6^ vs. Immun_Stim_Serpin_B9_rel_int |
| --- | --- | --- | --- |
| Spearman r |  |  |  |
| r | -0,01412 | -0,209 | -0,1383 |
| 95% confidence interval | -0,4439 to 0,4209 | -0,6054 to 0,2704 | -0,5571 to 0,3365 |
|  |  |  |  |
| P value |  |  |  |
| P (two-tailed) | 0,9503 | 0,3765 | 0,5608 |
| P value summary | ns | ns | ns |
| Exact or approximate P value? | Approximate | Approximate | Approximate |
| Significant? (alpha = 0.05) | No | No | No |
|  |  |  |  |
| Number of XY Pairs | 22 | 20 | 20 |
